# A scoping review of policies to encourage breastfeeding, healthy eating, and physical activity among rural people and places in the United States

**DOI:** 10.1186/s12889-024-19173-7

**Published:** 2024-08-09

**Authors:** M. Renée Umstattd Meyer, Bailey Houghtaling, Marilyn E. Wende, Khawlah H. Kheshaifaty, Haley Delgado, Stephanie A. Eze, Cassady Mecate, Rebekah Summerall Woodward, Randa Lopez Morgan, Kathy J. Krey

**Affiliations:** 1 Department of Public Health, Robbins College of Health and Human Sciences, Baylor University, Waco, TX USA; 2 Gretchen Swanson Center for Nutrition, Omaha, NE USA; 3 Department of Health Education and Behavior, College of Health and Human Performance University of Florida, Gainesville, FL USA; 4 School of Nutrition and Food Science, Louisiana State University Agricultural Center, Louisiana State University, Baton Rouge, LA USA; 5 LSU Libraries, Louisiana State University, Baton Rouge, LA USA; 6 School of Education, Baylor University, Waco, TX USA

**Keywords:** Systems, Environment, Remote, Small town, Disparities, Exercise, Nutrition, Health promotion, Surveillance, Measurement

## Abstract

**Background:**

Rural U.S. residents experience a disproportionate burden of diet and physical activity (PA) related chronic disease compared to urban residents, due to resource and economic challenges. Diverse policy approaches for chronic disease prevention have been implemented to address barriers to breastfeeding, healthy eating, and PA. Therefore, the purpose of this paper is to describe policy supports for breastfeeding, healthy eating, and/or PA occurring in rural U.S. areas.

**Methods:**

A scoping review was conducted March-June 2020 to identify policy, systems, and environment change approaches occurring in the rural U.S. for breastfeeding, healthy eating, and PA. Search procedures were guided by the PRISMA-ScR, Arksey and O’Malley’s work (2007), and a science librarian. Medline, PubMed, Web of Science, and Agricola were used to identify peer-reviewed research. ProQuest Dissertations and Theses A&I were used to identify dissertation research. Grey literature searches included Google, Google Scholar, government pages, and public health, federal nutrition assistance program, Cooperative Extension Services, and related webpages. Policy results are reported and inclusion criteria were: (1) breastfeeding, healthy eating, and/or PA focus; (2) about policy factors; (3) specific to U.S. rural populations/places; and (4) English language. Outcomes (study/source design, objective(s), methods/measurement, setting, population characteristics, behavioral focus, policy-specific results) were extracted into a standardized Excel document.

**Results:**

Results include 122 total sources: original research, with some sources referencing multiple behaviors, (*n* = 74 sources: 8 breastfeeding, 41 healthy eating, 42 PA), grey literature (*n* = 45 sources: 16 breastfeeding, 15 healthy eating, 27 PA), and graduate research *(n* = 3 sources: 1 breastfeeding, 2 healthy eating, 1 PA). Breastfeeding policy initiatives included policies or programs at hospitals, increasing access to resources, and improving culture or norms at workplaces. Healthy eating policy initiatives included increasing access to healthy foods, reducing financial burden, implementing programs, food assistance programs, and healthy food prescriptions at healthcare facilities. PA policy initiatives focused on Complete Streets, joint or shared use efforts, Safe Routes to Schools, master plans for greenways, trails, and/or transportation, school health plans, and childcare/school standards.

**Conclusions:**

Results from this scoping review compile and offer commentary on existing policy solutions to improve breastfeeding, healthy eating, and/or PA in the rural U.S.

## Background

Rates of chronic disease among Americans are high [1–3] and projected to increase over time [4]. This is important, since living with multiple chronic diseases is associated with greater health care use and cost (e.g., doctor office visits, prescriptions) [1]. For example, 90% of health care spending is accounted for by the 60% of Americans with at least one chronic disease and 41% accounted for by the 12% of Americans with five or more chronic diseases [1]. Additionally, experiencing multiple chronic conditions leads to increased risk of mortality [5]. Disparities also exist with respect to chronic disease morality. Rural compared to urban residents in the United States (U.S.) have higher mortality rates from all five leading causes of death, including cancer, heart disease, unintentional injury, chronic lower respiratory disease, and stroke [6, 7].

To address high rates of chronic disease and related disparities, chronic disease prevention must address behavioral risk factors on multiple levels of influence and across the entire lifespan [8]. National guidelines for the promotion of breastfeeding [9], healthy eating [10, 11], and physical activity (PA) [10, 12] indicate areas for improvement regarding U.S. population health behaviors for chronic disease prevention. However, education approaches alone are unlikely to favorably impact rural Americans’ health practices [13]. As such, concerted policy, systems, and environmental (PSE) changes to improve breastfeeding, healthy eating, and PA practices in alignment with guidelines in settings where Americans “live, learn work, shop, and play” are needed [14–18].

PSE changes may be especially impactful for rural U.S. populations who experience a higher burden of diet and PA related chronic disease, due to infrastructure, resource, and economic challenges [19–28]. For example, rural Americans are less likely than urban counterparts to initiate and sustain breastfeeding [29], to choose foods and beverages aligned with 2015–2020 Dietary Guidelines for Americans [11], or to meet PA guidelines [19, 20, 30, 31]. To mitigate rural health disparities, an understanding of PSE factors related to promoting breastfeeding, healthy eating, and/or PA in rural communities is needed. Further, understanding opportunities to track such factors over time can help move public health surveillance beyond individual-level behaviors to monitor PSE factors more likely to influence populations’ health-related choices [15].

Researchers applying PSE approaches have called for additional investigation and analysis of policy approaches surrounding the allocation of resources and funding to high-risk populations [14, 15]. Indeed, policy approaches to improve health behaviors among rural populations are studied as a means to implement empirically supported strategies on the national, state, county, or organizational levels [16–18]. While many systematic reviews have compiled evidence-based environmental approaches to health promotion [19–25, 32–37], fewer recent reviews have compiled evidence-based policy approaches to address low rates of breastfeeding, healthy eating, and PA that may have profound implications for population health outcomes [38]. Lastly, to our knowledge, no scoping reviews have been published on rural behavior change policy approaches that may be included within less formal channels, such as grey literature reports and theses.

There is a need to comprehensively review and compile existing policy approaches to address low rates of breastfeeding, healthy eating, and PA in rural areas. Scoping reviews can provide information for effective implementation and evidence-based policy strategies as chronic disease prevention strategies [39], and be important tools for compiling academic and non-academic sources to understand the breadth of policies that have been implemented in rural areas [39]. Given the above considerations, the purpose of this scoping review was to identify policy supports that encourage breastfeeding, healthy eating, and/or PA practices among rural American communities. A secondary aim was to assess policy measurement approaches used to collect data in rural communities.

## Methods

### Design

A broad scoping review was conducted in 2020 as part of a large project to inform PSE surveillance indicators for breastfeeding, healthy eating, and PA promotion among U.S. rural people and places. This project was contracted by the Division of Nutrition, PA, and Obesity Centers for Disease Control and Prevention (CDC). CDC partners aided in developing the review focus and provided feedback, although were not responsible for synthesizing results or drawing conclusions. Notably, the below methods section details the approach used for the entire review, although only the results pertaining to policy are presented here. Carrying out the scoping review procedures, as detailed below, resulted in the inclusion of over 300 sources that were described in an internal-facing report to CDC. To facilitate the reporting of results to a wider audience and improve the ability to offer specific recommendations, authors split results for breastfeeding, healthy eating, and PA by policy, systems, or environmental strategies.

Both the 2018 Preferred Reporting Items for Systematic Reviews and Meta-Analyses extension for Scoping Reviews (PRISMA-ScR) [40] and guidance published by Arksey and O’Malley [41] were used to inform the review strategy and reporting.

The review team included scholars with expertise in rural health and PSE change strategies to improve breastfeeding, healthy eating, and PA patterns/practices. A library partner, a team of graduate research assistants, and an expert advisory board were also involved. Advisory board members (*n* = 6) included well-established researchers in the areas of PSE and breastfeeding (*n* = 2), healthy eating (*n* = 2), or PA (*n* = 2), with experience working with rural communities when possible. Training was arranged for graduate research assistants at the start of the review process, covering topics including literature review methods, PSE examples, and data extraction. Findings related to policies to encourage/support rural breastfeeding, healthy eating, and PA patterns/practices are reported here and other results (i.e., systems, environments, and qualitative case studies) are reported separately (forthcoming). A review protocol was pre-registered using Open Science Framework (OSF; 10.17605/OSF.IO/VXMDC [42].

### Search strategy

A science librarian tested and selected the search strategy used to identify sources, designed to broadly capture policy, systems, and environmental strategies for breastfeeding, healthy eating, and PA promotion occurring in rural settings. Grey literature, graduate research (including thesis and dissertation work), and peer-reviewed scientific literature were all of interest. Searches occurred over a 5-month period between February and July 2020. The procedures for searches are described below by source type.

### Peer-reviewed scientific articles and graduate research

Four academic databases – Medline, PubMed, Web of Science, and Agricola – were selected to identify peer-reviewed research across the three discipline areas and ProQuest was used to identify graduate research. Given the broad focus of the scoping review, key terms were tested to select words that most accurately captured relevant sources without overly restricting search databases. Key terms were applied to databases between March and June of 2020 by one researcher with terms focused on topic area (e.g., breastfeed*, diet*, “physical activity*”), geography (e.g., rural*, “United States”), and setting (e.g., policy, environment*) (see 10.17605/OSF.IO/VXMDC for the full search strategy) [42].

The year 2000 was used as a search restriction for peer-reviewed research given PSE strategies for breastfeeding, healthy eating, and PA were less of a focus among the scientific community prior to this year [43]. ProQuest searchers were limited by topic area. For example, search restrictions for healthy eating and PA included only dissertation research and the year 2018 and, for breastfeeding, only the year 2015 was used due to fewer retrieved results compared to the other topic areas. Graduate research prior to these years were assumed to be published and thus would have been identified through the peer-reviewed literature searches. The complete search strategy is available at 10.17605/OSF.IO/VXMDC [42].

**Fig. 1:**
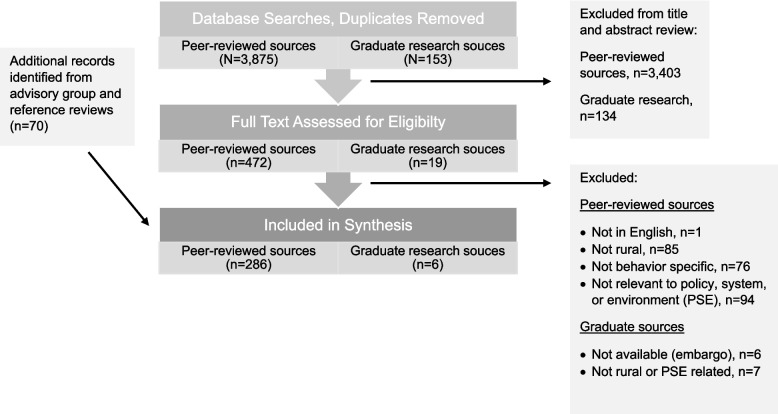
PRISMA diagram of included and excluded studies related to policy, systems, and environmental change approaches for breastfeeding, healthy eating, and physical activity promotion

All search results were downloaded to an EndNote X9 file for title and abstract review. Due to the large scope of the review, full text reviewing occurred independently among project team members. Figure 1 provides a PRISMA diagram of included and excluded studies related to all three PSE approaches, with reasons for exclusion. This process was iterative. Trained research team members (HD, SE, CM, RSW) completed abstract review, full text review, and extraction, with liberal inclusion of source materials, and then three project leads (BH, KJK, and MRUM) and trained research team members (KHK, MEW) checked eligibility of all extracted full text articles. The flow diagram regarding academic and ProQuest sources reviewed and included in our synthesis among all behaviors and PSE areas is shown in Fig. 1.

To supplement the systematic search, literature recommendations were solicited from the advisory group, including both original research and related systematic/scoping reviews. Research team members reviewed and determined if advisory group recommendations met inclusion criteria.

### Grey literature

Grey literature searches spanned Google, Google Scholar, Google government pages (inurl:gov) and public health, federal nutrition assistance program, Cooperative Extension Services (Extension – a nationwide educational network that addresses public needs by providing non-formal higher education and learning activities to farmers, ranchers, communities, youth, and families) [44], and other webpages (see 10.17605/OSF.IO/VXMDC) [42]. Search lists were generated for breastfeeding, healthy eating, and PA topic areas with some overlap as appropriate.

Graduate research assistants visited webpages, identified grey literature documents, and reviewed sources for relevant information. Using an iterative process, trained research team members (HD, SE, CM, RSW) completed a review of all grey literature sources (with liberal inclusion of source materials) and then three project leads (BH, KJK, and MRUM) and trained research team members (KHK, MEW) checked these standardized Excel spreadsheets for clarity/completeness and inclusion eligibility.

### Inclusion and exclusion criteria

All sources were required to meet five criteria for inclusion. Specifically: (1) a focus on at least one of three topic areas (breastfeeding, healthy eating, and/or PA); (2) about PSE factors (rather than individual behavioral or interpersonal-level approaches); (3) results specific to rural U.S. populations or places; and (4) English language publication. There were no restrictions on research design which resulted in the inclusion of both objective and subjective data. Given the number of sources identified, social environment factors were excluded after the search to narrow the review focus, as social factors (e.g., peer support, social marketing) were considered less consistent indicators for potential public health surveillance [45].

### Rural definition

Several definitions of “rural” are used in the literature and across organizations. For this review, rural settings were determined using the source description. For grey literature sources, the “Am I Rural” search tool was used to determine source inclusion if there was no clear description/classification of rural and a location (town, county) was reported. The “Am I Rural” search tool, which uses common rural definitions to provide rural classifications for certain locations, including Census definition (designating Urbanized areas and Urban Clusters), Core Based Statistical Areas, Federal Office of Rural Health Policy defined rural areas, Frontier and Remote Area (FAR) codes by census tract defined rural areas, Rural Urban Commuting Areas (RUCA) codes by census tract, Rural-Urban Continuum Codes (RUCC), and Urban Influence Codes (UIC) [46]. To reflect the heterogeneity of rural people and places and to address the inadequacy of standard rural definitions in capturing sociodemographic and cultural variations, study sites were characterized, when possible (county or town information listed), using a rural–urban typology for rural spaces, including: African American South; Aging Farmlands; Evangelical Hubs; Graying America; Hispanic Centers; Latter Day Saints Enclaves; Native American Lands; Rural Middle America; and Working Class Country [47, 48].

### Scoping review outcomes and results synthesis

Outcomes were extracted to standardized Excel sheets designed by study leads and reviewed by CDC partners and advisors, including the study or source design and objective, setting or sector, population characteristics, behavioral focus, and results specific to rural PSE factors. PSE change definitions were sourced from Supplemental Nutrition Assistance Program Education (SNAP-Ed, a federally-funded grant program that supports evidence-based nutrition and obesity prevention interventions and projects for those eligible for SNAP) [49] guidance due to the large organizational emphasis on using these types of strategies to improve community health outcomes. Thus, a “policy” was considered a “*written statement of an organizational position, decision, or course of action”*. All outcomes were extracted by multiple researchers using an iterative process.

## Results

Results from this scoping review include 122 total sources focused on policy, which are compiled in Table 1. Table 2 presents detailed information about original research (*n* = 73 sources: 8 breastfeeding, 41 healthy eating, and 41 PA, where *n* = 17 included 2 behaviors). Table 3 presents results specific to grey literature (*n* = 45 sources: 15 breastfeeding, 16 healthy eating, and 28 PA, where* n* = 12 included 2 behaviors and *n* = 1 included all 3). Table 4 presents results specific to graduate research (*n *= 3 sources: 1 breastfeeding, 2 healthy eating, and 1 PA, where *n *= 1 included 2 behaviors).

### Breastfeeding

Rural settings which cited breastfeeding policy in grey literature, graduate research, and peer-reviewed sources focused on initiatives in hospital/healthcare settings [50–68], workplaces [68–73], schools [68], food assistance programs [29, 74], libraries [72], and/or local business settings [72].

Breastfeeding policies in rural hospital and healthcare settings mainly focused on the implementation of the Baby-Friendly Hospital Initiative, CDC’s Ten Steps to Successful breastfeeding, High 5 for Mom & Baby, or similar practices to create healthcare norms and environments supportive of breastfeeding initiation after birth [51–55, 57, 59, 61, 62, 64–67]. A specific policy to encourage maternal-infant skin-on-skin contact immediately postpartum was found promising for encouraging rural breastfeeding initiation and duration. [60, 61, 63] Another study found rural hospitals could improve the implementation of policies to support breastfeeding compared to more urban settings, and higher breastfeeding rates were found among hospitals implementing several steps of Baby-Friendly Hospital Initiative Ten Steps in both urban and rural settings [62].

Policy initiatives were similar among rural workplaces, schools, libraries, and local business setting, and included leadership decisions that increase support and resources [70–73], and provide protocols for breastfeeding [69]. Examples of food assistance program policy changes included addressing inconsistency of breastfeeding promotion and practices [29]. and included benefits for breastfeeding women using food assistance (e.g., Special Supplemental Nutrition Program for Women, Infants, and Children) [29, 74].

Methods and tools used to assess rural breastfeeding policies included data from Baby Friendly USA [61, 64], surveys or questionnaires (e.g., Maternity Practices in Infant Nutrition and Care survey) [60, 62, 63, 70], and interviews (Table 5) [29, 71].

### Healthy eating

Rural settings focused on healthy eating policy involved initiatives in schools [75–106], food assistance programs [97, 101, 102, 107–114], food retail [98, 99, 101–103, 107, 109, 113, 115–121], childcare settings [122–125], healthcare settings [119, 121, 126, 127], local food producers [83, 109, 118, 121, 128], churches [91, 129], community gardens [130], neighborhoods [110, 131], health departments [132], local government [133], and workplaces [73, 95, 130, 133].

Policy initiatives in rural schools and childcare settings included promoting healthy food (e.g., advertisement, cafeteria monitors encouraging healthy choices) [82, 86, 88, 95, 103], adopting federal or state level child nutrition programs (e.g., farm to school programs, U.S. Department of Agriculture school meal programs) [78, 79, 83, 90, 93], prohibiting or limiting access to unhealthy foods at schools [76, 80, 87, 89, 95, 104–106, 115], allowing students to bring water bottles to school [91], enacting school breakfast programs [75, 81, 84, 86, 90, 100], increasing healthy food availability [75, 82, 85, 91, 94, 95, 100, 123], reversing lunch and recess [104–106], requiring health education (e.g. education in diabetes best practices) [76, 80], and adopting school nutrition standards [75, 76, 85, 92, 93, 125]. Barriers cited in school and childcare settings include lack of capacity or training to implement food programming [88, 105, 124], federally mandated academic testing requirements [80], lack of nutrition standards or ability to influence cafeteria foods [80], lack of access to healthy food outlets [94], cost of healthy foods or funding for healthy eating policy [87, 94, 98, 124], use of unhealthy foods as rewards [91], and inability of food service directors to implement healthy food policy [87].

Food assistance programs, such as the Federal Distribution Program for Indian Reservations, SNAP, and Women Infants and Children (WIC), promote health eating by addressing food insecurity, financial stress, and healthy food access in rural and urban communities. Food assistance programs were highlighted as vital for rural communities given higher rates of food insecurity and financial stress, and low access to healthy food among rural populations [97, 101, 102, 107–114]. Food assistance program policies that restrict the amount of and access to benefits [110], or those that do not improve access to healthy foods [111], were identified as potential barriers.

Rural food retail policy initiatives included promoting local produce using vouchers [119], food prescriptions [119], or advertisements [115, 116, 118, 120], reducing costs at healthy food stores [116], providing incentives for small, healthful food stores [117], creating co-operative services [107, 120], and changing healthful food store zoning [99]. Barriers to promoting healthy eating using food retail policy included food systems issues [98, 118], low access to healthy food stores [116], and low enforcement and implementation [99].

Healthcare setting policy initiatives in rural areas included sodium reduction policies (e.g., lower sodium options, fast food free zones) [127] and farmers market prescriptions/vouchers from physicians [119, 126, 127]. Barriers in healthcare settings included low implementation of or adherence to new policies among physicians [119], and lack of funding for new policy initiatives [127].

Additionally, policy initiatives for rural food producers, churches, community gardens, neighborhoods, health departments, local governments, and workplaces similarly focused on increasing access to healthy foods, increasing access to food preparation equipment, developing nutrition standards, garnering political support, implementing shared use agreements, and disseminating health promotion materials [73, 83, 91, 95, 109, 110, 118, 128–133].

Methods and tools used to assess rural healthy eating policies included interviews and/or focus groups [80, 87, 88, 98, 105, 109, 112, 119, 128–130, 132], surveys [83, 89, 91, 105, 106, 109, 113, 118, 127, 133, 134], assessment tools [82, 96, 98, 122–124, 131], administrative data [79, 84, 90, 116], policy coding forms [99], workshops [110], observational tools [92], photovoice [94], and process evaluation measures [86] (Table 5).

### Physical activity

Rural settings for identified policy results for PA included schools [75–77, 80, 82, 87, 88, 91, 93–95, 98, 100–104, 106, 107, 120, 125, 135–156], trails/sidewalks/paths [115, 130, 138, 149, 154, 155, 157–163], streets [100, 102, 103, 113, 125, 136–145, 155–159, 161, 162, 164–169], childcare [122–125, 162], recreation facilities [98, 101, 107, 113, 114, 134, 138, 140, 141, 144, 170, 171], churches [91, 129, 172], parks/playgrounds [75, 95, 98, 102, 103, 113, 114, 134, 141, 161], healthcare settings [126], county fairgrounds [95], workplaces [73, 95, 149, 161, 162], neighborhoods [110, 131, 163], commercial/retail outlets [141], and community gardens [130]. In addition, many studies noted PA policies that were not setting specific [94, 115, 131]. Policy initiatives for PA focused on Complete Streets efforts (i.e., an approach to planning, designing and building streets that enables safe access for all users) [103, 125, 164, 165, 167, 168], joint or shared use efforts with schools [75, 101, 120, 146] or churches [172], Safe Routes to Schools efforts [100, 125, 136, 137], and coordinated plans or master plans for the community or county (greenway, trail, town, pedestrian, bicycle, transportation) [100, 136, 141, 157, 160], a coordinated school health plan [100] or childcare PA standards [125].

In rural schools or childcare settings, policy supports for PA included joint/shared use efforts [75, 101, 120, 138, 139, 146–148, 152], designated walk/bike to school days or programs [100, 103, 125, 136, 137, 143, 144, 154], PA breaks during the school day (e.g., brain breaks, mini field trips, outside time) [123, 147, 150], after-school PA programming [107, 155], making changes to the recess period [104], integrating PA into classroom activities [80], PA curriculum [104], PA standards [125], supporting or requiring physical education (PE) in schools [76, 80, 98, 100, 150], and increasing PE time [104]. Policy barriers at schools included insufficient time for recess or PA [87, 88], teachers withholding PA as punishment [88], federally mandated academic testing requirements [80, 135], low population sizes in rural areas, distance to schools [153, 155], community perceptions that schools are “off limits” during after hours [148], limited recreational facilities [94, 153], lack of trained personnel/staff for implementation [124, 153], limited funding [87, 124, 135], absence of political support (from administrators or policymakers) [150], conflicts with other school activities [153], and limited formal PE requirements [87].

Policy supports for PA focused on rural streets, trails, sidewalks, and paths included bicycle and pedestrian plans [113, 125, 136, 138, 139, 141, 144, 145, 156, 159, 163–165, 167–169], ensuring compliance with the American Disabilities Act [161], enhancing or adding PA infrastructure [158, 163], which included ecotourism (e.g., facilitating connections with historical resources), pedestrian centered street improvements [113], maintenance of PA infrastructure [158], and addressed parks, schools, farmlands, and commercial/retail areas as part of the plan [100, 103, 113, 130, 136–145, 157–159, 162, 164–169]. In addition, one study specifically noted policies to address the needs of rural, diverse populations, such as minority groups, low-income groups, elderly, and people with disabilities [141]. Barriers to policy changes in streets include lack of or inadequately maintained sidewalks [140, 145, 162], lack of awareness of existing policies [169], conflicting evidence informing policy, absence of political support [130, 140], limited funding [115, 130], safety concerns (e.g., stray animals, lack of safety features, traffic) [115, 155, 162], and graffiti [100, 103, 113, 136–145, 157–159, 162, 164–169].

Policies in rural parks or recreational facilities included improving or maintaining PA environments [103], ensuring PA programming is available year-round [107], addressing the needs of diverse populations (e.g., minority groups, lower income groups, the elderly, and persons with disabilities) [141, 161], implementing shared use agreements [113, 170, 171], developing land use plans, and allowing public use of PA resources [114]. Potential barriers to PA policies included lack of funding, lack of availability of coalition members and zoning, more mixed-use zoning needed [140], tending to public and outdoor spaces (e.g., litter, issues with grass) [170], and differences in political agendas as a possible reason for varying priorities in PA-supportive environmental change (e.g., recreational facilities, sidewalks, mixed-use school athletic spaces) [113, 140, 141, 149, 170]. Park-specific barriers include distance to parks and inadequately maintained park amenities [113, 134, 140, 141, 161].

Finally, policy changes across rural faith-based organizations (churches), healthcare settings, county fairgrounds, workplaces, neighborhoods, commercial/retail outlets, and community gardens included requirement of social events to include youth PA opportunities, subsidizing gym memberships, and joint use agreements [73, 91, 95, 110, 126, 129–131, 162, 163, 172]. Barriers across other settings included lack of access to or absence of PA resources, funding instability, safety concerns, existing organizational practices, and distance to PA opportunities [91, 95, 126, 129–131, 172].

Methods and tools used to assess rural PA policies included interviews and/or focus groups [80, 87, 88, 104, 115, 129, 130, 135, 147, 150, 151, 155, 162, 171], surveys [104, 106, 113, 117, 131, 135, 149, 152, 153, 158, 159, 169, 170], assessment tools [122–124, 138, 139, 142, 144, 145, 155, 161], administrative data [93, 141], workshops [110], and photovoice [94] (see Table 5).

**Table 1:** Source Characteristics of breastfeeding, healthy eating, and physical activity policy efforts in rural U.S. settings (*n* = 122)

Source		Behavior			Setting				
Source Author	Source Type -Source Title	BF	HE	PA	Location	Census Region	Rural Typology	Rural Definition	Setting(s)
Ahmed, 2011 [80]	Original Research: Perceptions of middle school educators in Hawai'i about school-based gardening and child health		**X**	**X**	Hawaii, U.S	Pacific	Graying America	Authors described study setting as rural	Schools
Alaska Department of Health and Social Services, Division of Public Health, 2020 [50]	Grey Literature: Alaska Breastfeeding Initiative	**X**			Soldotna, Alaska	Pacific	Graying America	Met at least one criterion for rural using “Am I Rural?” tool	Hospitals
Allen, 2015 [63]	Original Research: Breastfeeding supportive hospital practices in the U.S. Differ by County urbanization level	**X**			Nationwide	Northeast, West, Midwest, and South	Unable to determine	Rural–Urban Continuum Codes (RUCC)	Hospitals
Amerson, 2014 [127]	Original Research: Adoption of sodium reduction strategies in small and rural hospitals, Illinois, 2012		**X**		Illinois	Midwest	Unable to determine	Authors described study setting as rural	Hospitals
Aytur, 2011 [141]	Original Research: Pedestrian and bicycle planning in rural communities: Tools for active living			**X**	North Carolina	South	Unable to determine	U.S. Census definition	Parks; recreational facilities; schools; streets
Baker, 2017 [147]	Original Research: Implementing and evaluating environmental and policy interventions for promoting physical activity in rural schools			**X**	Missouri	Midwest	Unable to determine	Authors described study setting as rural	Schools
Battista, 2014 [122]	Original Research: Improving the physical activity and nutrition environment through self-assessment (NAP SACC) in rural area childcare centers in North Carolina		**X**	**X**	Ashe County, Alleghany County, and Watauga County, North Carolina	South	Working Class Country, Graying America, College Towns	Small cities and rural areas were defined as those with populations less than 500,000	Childcare
Belansky, 2010 [105, 106, 135]	Original Research: Early & 5-year effects of the federally mandated Local Wellness Policy on school nutrition and PA environments appear modest in Colorado's rural, low-income elementary schools		**X**	**X**	Colorado	West	Unable to determine	Authors described study setting as rural	Schools
Belansky, 2013 [104]	Original Research: Adapted intervention mapping: A strategic planning process for increasing physical activity and healthy eating opportunities in schools via environment and policy change		**X**	**X**	Intermountain Valley region in south-central Colorado	West	Unable to determine	Authors described study setting as rural	Schools
Bishop, 2015 [75]	Grey Literature: Tucumcari HEAL MAPPS Community Report		**X**	**X**	Tucumcari, New Mexico	West	Graying America	Authors described study setting as rural	Playgrounds; schools
Calancie, 2017 [118]	Original Research: Food Policy council case study describing cross-sector collaboration for food system change in a rural setting		**X**		Adams County, Gettysburg, Pennsylvania	North East	Rural Middle America	Authors described study setting as rural	Local food producers; food retail
California Breastfeeding Coalition [66]	Grey Literature: Baby Friendly Hospital Initiative	**X**			Barstow, CA; King City, CA; Placerville, CA; Bishop, CA; Twentynine Palms, CA; Jackson, CA; Truckee, CA; Santa Paula, CA; Jackson, CA; Fort Irwin, CA	West	Urban Suburbs; Hispanic Centers; Exurbs; Graying America	Met at least one criterion for rural using “Am I Rural?” tool	Hospitals
Carlson, 2017 [169]	Original Research: Prevalence of complete streets policies in U.S. municipalities			**X**	U.S. municipalities	Northeast, Midwest, South, West	Unable to determine	2010 U.S. Census Bureau definition: municipalities with a population of 50% or less are rural	Streets
Carlton, 2017 [148]	Original Research: Shared use agreements and leisure time physical activity in North Carolina public schools			**X**	North Carolina	South	Unable to determine	National Center for Education Statistics system for determining rurality of schools	Schools
Case P, 2015 [172]	Grey Literature: Bonanza, Oregon HEAL MAPPS Community Report			**X**	Bonanza, Oregon	West	Graying America	Authors described study setting as rural	Churches
Caspi, 2017 [81]	Original Research: School Breakfast Program participation and rural adolescents' purchasing behaviors in food stores and restaurants		**X**		Minnesota	Midwest	Unable to determine	Authors described study setting as rural	Schools
Center for Health Equity, Education, and Research, 2017 [67]	Grey Literature: Mississippi Champs Hospital	**X**			Oxford, MS; Columbus, MS; Meridian, MS; New Albany, MS; Picayune, MS; Brookhaven, MS; Tupelo, MS; Amory, MS; Indianola, MS; McComb, MS	South	College Towns; African American South; Evangelical Hubs	Met at least one criterion for rural using “Am I Rural?” tool	Hospitals
Center for Health Equity, Education, and Research, 2017 [68]	Grey Literature: CHEER Champion of the Week: Blackfeet Nation Community Breastfeeding Team	**X**			Browning, Montana	West	Native American Lands	Met at least one criterion for rural using “Am I Rural?” tool	Hospitals; workplace
Center for Health Equity, Education, and Research, 2017 [51]	Grey Literature: Chippewa Cree Tribe Increases Support for Breastfeeding	**X**			Box Elder, MT; Havre, MT	West	Graying America	Met at least one criterion for rural using “Am I Rural?” tool	Hospitals
Chrisman, 2014 [149]	Original Research: Perceived correlates of domain-specific physical activity in rural adults in the Midwest			**X**	Iowa	Midwest	Unable to determine	A county with no principal city with a population of over 50,000 people (U.S. Department of Health and Human Services)	Trails; schools; workplace
Chrisman, 2015 [171]	Original Research: Environmental influences on physical activity in rural Midwestern adults: a qualitative approach			**X**	Iowa	Midwest	Unable to determine	A county with no principal city with a population of over 50,000 people (U.S. Department of Health and Human Services)	Recreation facilities
Cleary, 2018 [116]	Original Research: Store profitability and public policies to improve food access in non-metro U.S. counties		**X**		Nationwide	Northeast, West, Midwest, and South	Unable to determine	RUCC	Food retail
Demment, 2015 [82]	Original Research: Rural middle school nutrition and physical activity environments and the change in body mass index during adolescence		**X**	**X**	New York	Northeast	Unable to determine	Based on the National Center for Education Statistics	Schools
Drummond, 2009 [123]	Original Research: A pebble in the pond: the ripple effect of an obesity prevention intervention targeting the childcare environment		**X**	**X**	Yuma County, Yuma, Arizona	West	Hispanic Centers	Authors described study setting as rural	Childcare
Dumphy, 2016 [69]	Original Research: A Breastfeeding Quality Improvement Project in rural primary care	**X**			Georgia	South	Unable to determine	Authors described study setting as rural	Workplace
Evenson, 2011 [158]	Original Research: Planning for pedestrians and bicyclists: results from a statewide municipal survey			**X**	North Carolina	South	Unable to determine	Based on the 2006 U.S. Census, rural municipalities population of < 5000 persons	Trails; streets
Farris, 2014 [92]	Original Research: Nutritional comparison of packed and school lunches in pre-kindergarten and kindergarten children following the implementation of the 2012–2013 National School Lunch Program standards		**X**		Montgomery County and Giles County, Virginia	South	Rural Middle America	Authors described study setting as rural	Schools
Findholt, 2011 [94]	Original Research: Photovoice engages rural youth in childhood obesity prevention		**X**	**X**	Union County, LaGrande, Oregon	West	Graying America	Authors described study setting as rural	Schools
Flower, 2008 [29]	Original Research: Understanding breastfeeding initiation and continuation in rural communities: a combined qualitative/quantitative approach	**X**			North Carolina, Pennsylvania	South, Northeast	Unable to determine	Authors described study setting as rural	Food assistance programs
Foster, 2015 [124]	Original Research: Evaluation of nutrition and physical activity policies and practices in childcare centers within rural communities		**X**	**X**	Indiana; Kansas; Michigan; North Dakota; Ohio; South Dakota; Wisconsin	Midwest	Unable to determine	Authors described study setting as rural	Childcare
Fredericks, 2015 [101]	Grey Literature: Kalama, Washington HEAL MAPPSTM Community Report		**X**	**X**	Kalama, WA	West	Graying America	Authors described study setting as rural	Recreational facilities, schools, food assistance programs; food retail
Friedman, 2014 [119]	Original Research: Provider communication and role modeling related to patients' perceptions and use of a federally qualified health center-based farmers' market		**X**		South Carolina	South	Unable to determine	Author describes study setting as rural	Healthcare; food retail
Friesen, 2010 [95]	Original Research: Operation wellness: A university/community collaboration to enhance adult wellness		**X**	**X**	Indiana	Midwest	Unable to determine	Author describes study setting as rural	County fair; parks; schools; workplace
Foundation for Healthy Communities, 2016 [125]	Grey Literature: Celebrating our Collective Progress: 10 years of healthy eating and active living in New Hampshire		**X**	**X**	New Hampshire	Northeast	Unable to determine	Author describes study setting as rural	Schools; streets; childcare
Gamble, 2017 [150]	Original Research: Not enough time in the day: A qualitative assessment of in-school physical activity policy as viewed by administrators, teachers, and students			**X**	Mississippi	South	Unable to determine	Authors described study setting as rural	Schools
Glagola Dunn, 2018 [129]	Graduate Research: Examining Faith Based Communities as Leverage Points for The Prevention of Childhood and Adolescent Obesity [Dissertation]		**X**	**X**	Columbia, South Carolina	South	African American South	Authors described study setting as rural	Churches
Halverson, 2015 [102]	Grey Literature: Molalla HEAL MAPPSTM Community Report 2015		**X**	**X**	Molalla, OR	West	Exurbs	Authors described study setting as rural	Charitable food system; community centers; food retail; parks; schools; streets
Harden, 2015 [114]	Grey Literature: Clatskanie HEAL MAPPS™ Community Report 2015		**X**	**X**	Clatskanie, OR	West	Rural Middle America	Authors described study setting as rural	Food assistance programs; parks; recreation facilities
Harden, 2015 [120]	Grey Literature: Rainier HEAL MAPPS Community Report		**X**	**X**	Rainier, Oregon	West	Rural Middle America	Authors described study setting as rural	Schools; food retail
Hafoka, 2017 [142]	Original Research: Assessing the active living environment in three rural towns with a high proportion of Native Hawaiians and other Pacific Islanders			**X**	Hawaii	Pacific	Unable to determine	Authors described study setting as rural	Schools, streets
High5 For Mom & Baby [53]	Grey Literature: High 5 for mom & baby facilities	**X**			Colby, KS; Hays, KS; Ottawa, KS; Pratt, KS	Midwest	Rural Middle America; College Towns	Met at least one criterion for rural using “Am I Rural?” tool	Hospitals
Hill, 2008 [76]	Grey literature—Building a Successful Community Coalition–University Partnership at the Arizona–Sonora Border		**X**	**X**	Douglas, Arizona	West	Military Posts	Authors described study setting as rural	Schools
Hill, 2016 [170]	Original Research: Do the features, amenities, and quality of physical activity resources differ between city and county areas of a large rural region?			**X**	Dan River region, Virginia and North Carolina	South	African American South	Rural Urban Community Area (RUCA) codes	Recreation facilities
Holston, 2020 [103]	Grey Literature: Implementing policy, systems, and environmental change through community coalitions and extension partnerships to address obesity in rural Louisiana		**X**	**X**	Madison, Tensas, and St. Helena counties, Louisiana	South	African American South	Authors described study setting as rural	Streets; food retail, parks, school
Horne, 2013 [130]	Original Research: Implementing the ACHIEVE model to prevent and reduce chronic disease in rural Klickitat County, Washington		**X**	**X**	Klickitat County, Washington	West	Graying America	Authors described study setting as rural	Community gardens; trails; workplace
Indiana (IN) State Department of Health [52]	Grey Literature	**X**			Indiana	Midwest	Rural Middle America	Met at least one criterion for rural using “Am I Rural?” tool	Hospitals
Ishdorj, 2020 [74]	Grey Literature: Are rural infants benefiting from WIC food package rule changes? Breastfeeding and infant feeding behaviors	**X**			38 states, two U.S. Districts & Territories, and ten Indian/Tribal Organizations (ITOs)	Unable to determine	Unable to determine	RUCC	Food assistance programs
Izumi, 2006 [83]	Original Research: Results from the 2004 Michigan Farm-to-School Survey		**X**		Michigan	Midwest	Unable to determine	Author described study setting as rural	Local food producers; schools
Jantzer, 2018 [70]	Original Research: Breastfeeding support in the workplace: The relationships among breastfeeding support, work-life balance, and job satisfaction	**X**			State unknown	Unable to determine	Unable to determine	Author described study setting as rural	Workplace
Jernigan, 2012 [128]	Original Research: Addressing food insecurity in a Native American reservation using community-based participatory research		**X**		Mendocino County, Northern California	West coast	Graying America	Author described study setting as rural	Local food producers
Jilcott Pitts, 2013 [115]	Original Research: Addressing rural health disparities through policy change in the stroke belt		**X**	**X**	Lenoir County, North Carolina	South	African American South	Author described study setting as rural	Food retail; trails
Jilcott Pitts, 2015 [131]	Original Research: Associations between neighborhood-level factors related to a healthful lifestyle and dietary intake, physical activity, and support for obesity prevention polices among rural adults		**X**	**X**	Lenoir County, North Carolina	South	African American South	Authors described study setting as rural	Neighborhoods
Kansas Breastfeeding Coalition, Inc. [54]	Grey Literature: Communities supporting breastfeeding	**X**			Hutchinson, KS; Salina, KS	Midwest	Rural Middle America	Met at least one criterion for rural using “Am I Rural?” tool	Childcare
Kearny County Hospital [121]	Grey Literature: Fast Facts: A community report on your investment in Kearny County Hospital		**X**		Kearny County, KS	Midwest	Hispanic centers	Met at least one criterion for rural using “Am I Rural?” tool	Food retail, local food producers, hospitals
LaJeunesse, 2019 [143]	Original Research: Diverse school community engagement with the North Carolina active routes to school project: a diffusion study			**X**	Appalachian region, North Carolina	South	Unable to determine	Rural counties were those with average population densities of 250 people or less per square mile	Schools; streets
Lange, 2019 [117]	Original Research: Local government retail incentives for healthier food retailers in the USA, 2014		**X**		Nationwide	West, Midwest, South, and Northeast	Unable to determine	Rural was any setting not defined as urban based on the 2010 U.S. Census Urban Area to Place Relationship File	Food retail
Larson, 2018 [84]	Original Research: A Low-Cost, Grab-and-Go Breakfast Intervention for Rural High School Students: Changes in School Breakfast Program Participation Among At-Risk Students in Minnesota		**X**		Minnesota	Midwest	Unable to determine	Authors described study setting as rural	Schools
Liberty, 2019 [61]	Original Research: A Geospatial Analysis of the impact of the Baby-Friendly Hospital Initiative on breastfeeding initiation in North Carolina	**X**			North Carolina	South	Unable to determine	RUCC	Hospitals
Lillehoj, 2012 [62]	Original Research: Implementation of the baby-friendly hospital initiative steps in Iowa hospitals	**X**			Iowa	Midwest	Unable to determine	Authors described study setting as rural	Hospitals
Lillehoj, 2016 [161]	Original Research: Prevalence of physical activity policies and environmental strategies in communities and worksites: The Iowa Community Transformation Grant			**X**	Iowa	Midwest	Unable to determine	Counties without a metropolitan area were considered rural based on the White House Office of Management and Budget (OMB) definition	Sidewalks; streets; parks; workplace
Lo, 2017 [140]	Original Research: Environmental influences on physical activity among rural adults in Montana, United States (U.S.): Views from built environment audits, resident focus groups, and key informant interviews			**X**	Montana	West	Unable to determine	RUCA codes 7 or higher	Facilities; recreation; streets; schools
Louisiana Department of Health, 2020 [55]	Grey Literature: The Gift. Gift designated facilities	**X**			Vivian, LA; Bogalusa, LA; Fort Polk South, LA; De Ridder, LA; Leesville, LA; Minden, LA; Bastrop, LA	South	African American South, Military Posts, Evangelical Hubs	Met at least one criterion for rural using “Am I Rural?” tool	Hospitals
Majee, 2016 [71]	Original Research: Four years later: Rural mothers' and employers' perspectives on breastfeeding barriers following the passage of the Affordable Care Act	**X**			Missouri	Midwest	Unable to determine	Authors described study setting as rural	Workplace
Mann, 2017 [85]	Original Research: Smart Snacks in School Standards in Appalachian Virginia Middle Schools		**X**		Virginia	South	Unable to determine	Authors describe study setting as rural	Schools
Martin, 2018 [79]	Graduate Research: The Role of Programs and Policies in Shaping the Observed School Nutrition Environment and Rural Childhood Obesity [Thesis]		**X**		South Dakota	Midwest	Unable to determine	RUCC 8 and 9	Schools
Mayo, 2013 [99]	Original Research: Associations between county and municipality zoning ordinances and access to fruit and vegetable outlets in rural North Carolina, 2012		**X**		North Carolina	South	Urban Typology, Graying America, Evangelical Hubs, African American South	Based on population size of the U.S. Department of Commerce, 2010 Census Urban and Rural Classification and Urban Area Criteria	Food retail
Meendering, 2016 [96]	Original Research: Bigger / = Better: The Comprehensiveness and Strength of School Wellness Policies Varies by school district size		**X**		Illinois	Midwest	Unable to determine	Authors describe study setting as rural	Schools
Menking-Hoggatt, 2017 [60]	Graduate Research: The Effect of Early Skin to Skin Contact on Breastfeeding Duration and Exclusivity: a Mixed Methods Study	**X**			West Virginia	South	Unable to determine	Authors described study setting as rural	Hospitals
Merewood, 2005 [64]	Original Research: Breastfeeding rates in U.S. Baby-Friendly hospitals: results of a national survey	**X**			Nationwide	West, Midwest, South, and Northeast	Unable to determine	Authors described study setting as rural	Hospitals
Metos, 2007 [93]	Original Research: The strength of school wellness policies: One state's experience		**X**	**X**	Utah	West	Unable to determine	Authors described study setting as rural	Schools
Missouri Department of Health and Senior Services, 2020 [65]	Grey Literature: “Show-me-5” Initiative	**X**			Hannibal MO; Marshall, MO; Osage Beach, MO; Lebanon, MO; Branson, MO; Poplar Bluff, MO; Chillicothe, MO	Midwest	Rural Middle America; Evangelical hubs; Graying America;	Met at least one criterion for rural using “Am I Rural?” tool	Hospitals
Moore, 2010 [151]	Original Research: A qualitative examination of perceived barriers and facilitators of physical activity for urban and rural youth			**X**	North Carolina	South	Unable to determine	Less than 1000 persons/square mile and lacking an urbanized area of 50,000 persons or more based pm the 2000 U.S. Census definition	Schools
Moore, 2017 [113]	Original Research: Development and implementation of a local government survey to measure community supports for healthy eating and active living		**X**	**X**	California and Minnesota	West, Midwest	Unable to determine	Authors described study setting as rural	Food assistance programs; food retail; recreation facilities; streets; parks
Morshed, 2015 [97]	Original Research: Effect of WIC food package changes on dietary intake of preschool children in New Mexico		**X**		New Mexico	West	Unable to determine	Authors described study setting as rural	Schools; WIC assistance programs
Mucioki, 2018 [109]	Original Research: Thinking inside and outside the box: local and national considerations of the Food Distribution Program on Indian Reservations (FDPIR)		**X**		Southern Oregon to Northern California	West	Urban Typology, Graying America	Authors described study setting as rural	Food assistance programs; food retail; local food producer
Nanney, 2019 [86]	Original Research: A group randomized intervention trial increases participation in the School Breakfast Program in 16 rural high schools in Minnesota		**X**		Minnesota	Midwest	Unable to determine	Authors described study setting as rural	Schools; school breakfast program
National Institute for Children’s Health Quality, 2020 [56]	Grey Literature: Being small has advantages for hospitals implementing the ten steps	**X**			Batavia, NY	Northeast	Rural Middle America	Authors described study setting as rural	Hospitals
National Physical Activity Society, 2016 [154]	Grey Literature: Stories from Small Towns: Hebron, Nebraska			**X**	Hebron, Nebraska	Midwest	Aging Farmlands	Met at least one criterion for rural using “Am I Rural?” tool	Schools; trails
National Physical Activity Society, 2016 [165]	Grey Literature: Stories from Small Towns: Soap Lake, Washington			**X**	Soap Lake, Washington	West	Hispanic Center	Met at least one criterion for rural using “Am I Rural?” tool	Streets
New Mexico (NM) Breastfeeding Task Force, 2019 [57]	Grey Literature: Baby-Friendly Hospitals & Clinics	**X**			Gallup, NM; Alamogordo, NM; Silver City, NM; Shiprock, NM; Ruidoso, NM; Clovis, NM; Rio Rancho, NM; Socorro, NM; Zuni, NM; Espanola, NM	West	Graying America; Military Posts; Native American Lands; Working Class Country; Exurbs; Hispanic Centers	Met at least one criterion for rural using “Am I Rural?” tool	Hospitals
Ndirangu, 2007 [110]	Original Research: Conducting needs assessment using the comprehensive participatory planning and evaluation model to develop nutrition and physical activity interventions in a rural community in the Mississippi delta		**X**	**X**	Louisiana, Arkansas, and Mississippi	South	Unable to determine	Authors described study setting as rural	Food assistance programs
Onufrak, 2016 [133]	Original Research: Nutrition standards for Food Service Guidelines for foods served or sold in municipal government buildings or worksites, U.S., 2014		**X**		Nationwide	Northeast, Midwest, South, and West	Unable to determine	Based on the 2007 U.S. Census Bureau and Census of Governments, which excluded municipalities that were classified as urban	Local government; workplace
Omura, 2017 [152]	Original Research: Shared use agreements between municipalities and public schools in the, 2014			**X**	Nationwide	Northeast, Midwest, South, West	Unable to determine	The 2010 U.S. Census Urban Area to Place Relationship File defines rural with ≤ 50% urban	Schools
Park, 2018 [134]	Original Research: Community-based policies and support for free drinking water access in outdoor areas and building standards in U.S. municipalities			**X**	Nationwide	Northeast, Midwest, South and West	Unable to determine	Used a ratio of urbanized area to total area within a municipality to classify urban/rural. Data derived from the 2012 U.S. Census	Recreational facilities; parks; playgrounds
Perry, 2015 [139]	Original Research: Active living environment assessments in four rural Latino communities			**X**	Central Washington	West	Hispanic Centers	Author describes study setting as rural	Schools; streets
Peterson, 2018 [159]	Original Research: Prevalence of master plans supportive of active living in U.S. municipalities			**X**	Nationwide	South, Northeast, Midwest, West	Unable to determine	According to the 2010 U.S. Census Bureau, municipalities with 50% or less living in rural areas are categorized as rural	Sidewalks; streets; trails
Pollack Porter, 2019 [166]	Grey Literature: Promoting physical activity with temporary street closures			**X**	Maryland, North Carolina, Oklahoma and Texas	South	Unable to determine	Author describes study setting as rural	Streets
Powers-Hammond, 2015 [146]	Grey Literature: Connell HEAL MAPPS Community Report			**X**	Connell, Washington	West	Hispanic Centers	Author describes study setting as rural	Schools
Prevention Institute, 2019 [156]	Grey Literature: Northeast Iowa Food & Fitness Initiative			**X**	Decorah, Iowa	Midwest	Rural Middle America	Author describes study setting as rural	Schools; streets
Reis-Reilly, 2018 [72]	Grey Literature: Breastfeeding in the community: Addressing disparities through policy, systems, and environmental change interventions	**X**			Green, KY; Gadsden, FL	South	Evangelical Hubs; Hispanic Centers	Author describes study setting as rural	Workplace; libraries; local businesses
Riley-Jacome, 2010 [153]	Original Research: Enhancing community capacity to support physical activity: the development of a community-based indoor-outdoor walking program			**X**	Albany, Columbia and Greene counties, New York	Northeast	Rural Middle America, Graying America	Author describes study setting as rural	Schools
Robert Wood Johnson Foundation, 2013 [100]	Grey Literature: Manistique, MI: 2013 RWJF Culture of Health Prize		**X**	**X**	Schoolcraft, Michigan	Midwest	College Town	Population of 4000 residents	Streets; schools
Robert Wood Johnson Foundation, 2017 [163]	Grey Literature: Garrett County, Maryland: A Community Holding Hands to Bridge its Divide			**X**	Garrett County, Maryland	South	Working Class Country	Author describes study setting as rural	Sidewalks, trails, neighborhoods
Robert Wood Johnson Foundation, 2018 [160]	Grey Literature: Hardworking Rural Community Taps a Deep Well of Hope			**X**	Klamath County, Oregon	West	Unable to determine	Author describes study setting as rural	Trails
Robert Wood Johnson Foundation, 2019 [126]	Grey Literature: Hearing from everyone on health		**X**	**X**	Columbia Gorge region in Oregon and Washington	West	Hispanic Centers	75,000 residents	Healthcare
Robinson, 2014 [138]	Original Research: Assessing environmental support for better health: Active living opportunity audits in rural communities in the southern U.S			**X**	Bullock, Al; Perry, Al; Sumter, Al; Wilcox, Al; Grenada, MS; Humphreys, MS; Panola, MS; Yazoo, MS	South	Evangelical Hubs; African American South	Author describes study setting as rural	Recreation facilities; schools sidewalks; streets
Rural Health Information Hub, 2018 [58]	Grey Literature: Strengthening the workforce to improve pregnancy outcomes in rural areas	**X**			Madison, Wisconsin	Midwest	College Towns	Author describes study setting as rural	Hospitals
Rural Health Information Hub, 2019 [77]	Grey Literature: Albert Lea Blue Zones Project		**X**	**X**	Albert Lea, Minnesota	Midwest	Rural Middle America	Author describes study setting as rural	Schools
Rural Health Information Hub, 2018 [136]	Grey Literature: Coordinated Approach to Child Health in the Upper Peninsula (CATCH-UP)			**X**	Baraga, Gogebic, Houghton, Keweenaw, and Ontonagon Counties, Michigan	Midwest	Rural Middle America	Met at least one criterion for rural using “Am I Rural?” tool	Schools; streets; CATCH-UP program
Rural Health Information Hub, 2020 [157]	Grey Literature: Granville greenways walkable community			**X**	Granville, North Carolina	South	African American South	Author describes study setting as rural	Streets; trails
Safe Routes Partnership, 2015 [137]	Grey Literature: Rural communities: Making safe routes work			**X**	Fairfax, Virginia	South	Urban Suburbs	Author describes study setting as rural	Schools; streets
Sánchez, 2014 [87]	Original Research: School wellness policy implementation: insights and recommendations from two rural school districts		**X**	**X**	Northern New Mexico	West	Unable to determine	Author describes study setting as rural	Schools
Sanderson, 2002 [162]	Original Research: Environmental, policy, and cultural factors related to physical activity among rural, African American women			**X**	Wilcox county, Alabama	South	African American South	Author describes study setting as rural	Childcare; sidewalks; streets; workplace
Schetzina, 2009 [88]	Original Research: Developing a coordinated school health approach to child obesity prevention in rural Appalachia: results of focus groups with teachers, parents, and students		**X**	**X**	Tennessee	South	Unable to determine	Author describes study setting as rural	Schools
Shah, 2019 [132]	Original Research: Leaders' Experiences in planning, implementing, and evaluating complex Public Health Nutrition Interventions		**X**		California	West	Unable to determine	Centers for Disease Control and Prevention. NCHS Urban–Rural Classification Scheme for Counties	Health department
Sharkey, 2008 [111]	Original Research: Neighborhood socioeconomic deprivation and minority composition are associated with better potential spatial access to the ground-truthed food environment in a large rural area		**X**		Brazos valley, Texas	South	African American South; Hispanic Centers; Working Class country	U.S. Census Summary File, 2000 “5 urban clusters (UC; population > 2,500), several smaller towns (population 156–1,555), and many unincorporated areas. The rural region covered a land area of 11,567 km2(258 km2 in 25 UC CBG) and was home to 119,654 people	Food retail
Sherry, 2008 [89]	Original Research: An Evaluation of elementary school nutrition practices and policies in a Southern Illinois County		**X**		Illinois	Midwest	Unable to determine	Author describes study setting as rural	Schools
Smart Growth America, 2017 [164]	Grey Literature: The Best Complete Streets Initiatives of 2017			**X**	Warsaw, Missouri	Midwest	Graying America	Met at least one criterion for rural using “Am I Rural?” tool	Streets
Smart Growth America, 2020 [167]	Grey Literature: Complete streets work in rural communities			**X**	Nationwide, with specific examples described from: Woodstock, NY; De Soto, MO; Manistique, MI	Northeast, West, Midwest	College Towns; Exurbs	Author describes study setting as rural	Streets
Smith, 2009 [112]	Original Research: Rural food deserts: Low-income perspectives on food access in Minnesota and Iowa		**X**		Minnesota and Iowa	Midwest	Unable to determine	1993 Urban Influence Code of 7, 8, or 9	Food assistance programs
Tah, 2019 [59]	Grey Literature: Four IHS hospitals complete baby-friendly re-designation	**X**			Claremore, OK; Belcourt, ND; Zuni, NM	Southwestern	Exurbs; Native American Lands; Zuni, NM	Met at least one criterion for rural using “Am I Rural?” tool	Hospitals
Thomson, 2019 [144]	Original Research: Assessment of town and park characteristics related to physical activity in the Lower Mississippi Delta			**X**	Lower Mississippi Delta	South	Unable to determine	Authors described study setting as rural	Schools; streets; recreation facilities
Thomson, 2019 [145]	Original Research: Assessment of neighborhood street characteristics related to physical activity in the Lower Mississippi Delta			**X**	Lower Mississippi Delta	South	Unable to determine	Authors described study setting as rural	Schools; streets
Tingey, 2015 [107]	Grey Literature: *Wells, NV HEAL MAPPS Community Report*		**X**	**X**	Wells, NV	West	Graying America	Authors described study setting as rural	Food assistance program, food retail, recreation facilities, schools
Turner, 2019 [90]	Original Research: Community eligibility and other provisions for universal free meals at school: Impact on student breakfast and lunch participation in California public schools		**X**		California	West	Unable to determine	Author described study setting as rural	Schools
U.S. Department of Agriculture, 2008 [78]	Grey Literature: The Pennsylvania SFSP rural area eligibility pilot evaluation		**X**		Pennsylvania	Northeast	Unable to determine	Author described study setting as rural	Schools
U.S. Department of Agriculture, 2008 [108]	Grey Literature: Report to congress: The Nebraska rural area eligibility determination pilot for the Child and Adult Care Food Program		**X**		Nebraska	Midwest	Unable to determine	Author described study setting as rural	Food assistance program
University of Missouri Extension, 2018 [73]	Grey Literature: Missouri Workplace Wellness Award	**X**	**X**	**X**	Missouri	Midwest	Unable to determine	Did not meet at least one criterion for rural using “Am I Rural?” tool	Workplace
Voices for Healthy Kids. American Heart Association, 2020 [168]	Grey Literature: Strong Coalition Enabled City of Pryor Creek to Pass First Complete Streets Ordinance in Oklahoma			**X**	Pryor, Oklahoma	South	Evangelical Hubs	Met at least one criterion for rural using “Am I Rural?” tool	Streets
Wallace, 2019 [91]	Original Research: Community Coalitions for Change and the Policy, Systems, and Environment Model: A community-based participatory approach to addressing obesity in rural Tennessee		**X**	**X**	Tennessee	South	Unable to determine	Authors described study setting as rural	Churches; schools
West, 2013 [98]	Original Research: Stakeholder perceptions of obesity-prevention strategies: a comparison of geographically diverse rural counties		**X**	**X**	North Carolina	South	Unable to determine	Authors described study setting as rural	Food retail; schools; parks; recreational facilities
Yousefian, 2009 [155]	Original Research: Active living for rural youth: Addressing physical inactivity in rural communities			**X**	Maine	Northeast	Unable to determine	RUCA codes 7, 10.4, and 10.5	Schools; streets; sidewalks

*U.S.* United States, *BF* Breastfeeding, *HE* Healthy Eating,* PA *Physical Activity

**Table 2:** Original research about breastfeeding, healthy eating, and physical activity policies in rural U.S. settings (*n*=73)

Source				Behavior			Results
First Author, Year (citation)	Sample Characteristics	Data Collection Measure/Method	Setting(s)	BF	HE	PA	Policy
Ahmed, 2011 [80]	2 administrators, 4 teachers, and 3 garden staff	Semi-structured interviews with questions about children’s health, health consequences of obesity, challenges to children’s nutrition/ health, and the effects of school gardening on children’s health and development	Schools		**X**	**X**	Policies to improve child health such as a school policy removing unhealthy foods from campus were discussed and believed to have resulted in improved classroom behavior and concentration. Federally mandated academic testing requirements were a barrier to health, nutrition, and physical education (PE), as resources for these were sacrificed to “teaching to the tests.” A lack of ability to influence the school cafeteria, which served high sugar breakfasts and unpalatable lunches, was another perceived barrier to improving nutrition
Allen, 2015 [63]	NA	Maternity Practices in Infant Nutrition and Care Survey that assessed infant feeding maternity care policies and practices, including 7 domains of care: Labor and delivery; feeding of breastfed infants; breastfeeding assistance; contact between mother and infant; facility discharge care; staff training; and structural and organizational aspects	Hospitals	**X**			Scores were lower among more rural hospitals for feeding of breastfed infants. From 2007 to 2011, the average Maternity Practices in Infant Nutrition and Care (mPINC) score increased from 54 to 65 among more rural hospitals. The urban–rural gap in mPINC scores decreased from 10 points in 2007 to 6 points in 2011 (although still 9 points lower than urban sites)
Amerson, 2014 [127]	9 funded hospitals and 21 unfunded hospitals in rural small hospitals	A web-based survey for identifying sodium reduction strategies. Informed by Under Pressure guidance or developed by hospitals	Hospitals		**X**		Funded hospitals were implementing 16 sodium reduction strategies per hospital (e.g., lower-sodium food options at points of purchase, fast-food free zones), on average, before the initiative and 27 at the conclusion of the funding period. Unfunded hospitals sodium reduction activities remained, on average, at 16 initiatives
Aytur, 2011 [141]	543 municipalities; 265 (48.8%) were rural (8 or 3%) implemented pedestrian/ bicycle plans; 168 (30.9%) were rural farmland (9 or 5.4%) implemented pedestrian/ bicycle plans; 55 total municipalities with implemented pedestrian/ bicycle. plans. Rural farmland income: 76 (14%) municipalities had rural, income < $30,000 (4 or 5.3% implemented pedestrian/ bicycle plans); 92 (16.9%) rural, income ≥ $30,000 (5 or 5.4% implemented pedestrian/ bicycle plans); 98 (18.1%) rural and higher non-white (6 or 6.1% implemented pedestrian/ bicycle plans); 70 (12.9%) rural and lower non-white (3 or 4.3% implemented pedestrian/bicycle plans); 69 (12.7%) were classified as rural farmlands by rural and ≥ median graduation rate (3 or 4.3% implemented pedestrian/ bicycle plans); 99 (18.2%) rural and < median graduation rate (6 or 6.1%) implemented pedestrian/ bicycle plans	North Carolina U.S. Census data was used to collect information about municipalities to compare between those that did and did not have pedestrian/bicycle plans and assess the relationship between rurality, socioeconomic characteristics	Parks; recreational facilities; schools; streets			**X**	Lower-income areas with rural farmland had the lowest prevalence of pedestrian or bicycle plan. Rural plans scored higher than urban plans on several elements of plan quality: public participation and planning processes, analysis of current trends and conditions, and implementation. Compared to urban areas, rural areas were more likely to document the inclusion of steering committees, land use planners, local transportation planners, engineering/public works departments, private sector/business groups, and economic development nonprofits as stakeholders in the planning process. Rural areas were less likely to document involvement of state transportation planners, public health professionals, and law enforcement officials. 75% of plans in areas with rural farmland cited goals to improve public health; 63% of plans in areas with rural farmland cited goals to encourage PA for transportation. More than half of all rural plans cited goals to support walkable communities, active living, or active community environments. Rural plans cited ecotourism, encouraging walking to/from local businesses and linking visitors to historical resources as motivating factors for plan development; ≥ 85% of plans discussed intersection treatments (e.g., curb cuts, signals, crosswalks, midblock crossing, median refuges, bulb-outs) and pedestrian wayfinding aids such as signals, signs, and markers; and ≥ 75% proposed streetscape improvements. When describing land uses, all the rural plans discussed parks, schools, and commercial/retail areas; 75% discussed employment centers, and 62% discussed residential areas. Half (50%) of rural plans proposed policies to address the needs of special populations (e.g., minority groups, lower income groups, the elderly, and persons with disabilities)
Baker, 2017 [147]	4 elementary schools included 5 administrators and 4 teachers	Qualitative interviews and SOPLAY observations to assess facilitators, barriers and the effect of the dissemination of environmental and policy changes on students’ behaviors	Schools			**X**	Brain breaks policy interventions increased PA among rural youth and helped students stay focused and engaged. The development of the walking tracks provided an opportunity for the teachers and administrators to reflect on some of the challenges in providing the community with use of facilities after school hours
Battista, 2014 [122]	29 childcare centers in four Counties of North Carolina. Total population = 9,656, 401. Population below poverty level = 15.5%; white, non-Hispanic = 65%	Pre-post test evaluation used the Nutrition and Physical Activity Self-Assessment for Child Care to assess compliance for nutrition and PA recommendations and standards in addition to workshops and goal settings	Childcare		**X**	**X**	Post childcare staff intervention, that included workshops and goal settings, more effective policies were in place to meet nutrition and PA standards. Childcare centers associated with school districts showed less improvement
Belansky, 2010 [105, 106, 135]	45 elementary schools; students who received free or reduced lunch rates ranged from 40 to 82% (mean 54.4%); ethnicity ranged from 0 to 72% Hispanic (mean 27%).; (*n* = 18 schools participated in interviews.)	Principals, foodservice managers, and PE teachers completed the School Environment and Policy Survey to assess healthy eating and PA related to environment and policy features of schools, being asked to categorize policies regarding the presence and enforcement of policies about school policies and factors related to PA and food, nutrition services, and PE and other PA programs, respectively Key informant interviews (*n *= 18) for nutrition were conducted with food services managers. PA-related questions were asked of principals and district-level staff responsible for the Local Wellness Policy. The purpose was to assess knowledge and familiarity with their district’s policy, degree of implementation, and barriers and facilitators to implementation and enforcement	Schools		**X**	**X**	At 5-years, minutes for PE and recess did not increase, nor did offerings of fresh fruits and vegetables, but schools adopted more policies prohibiting the removal of recess as a punishment or to be used to make-up missed instructional time/tests. More schools scheduled recess before lunch and developed food policies for parties and vending machines. At 1-year, food service managers did not believe the local wellness policies affected the nutritional content of their school meals. There were no significant changes in daily offerings of vegetables, schools with relationships with farmers markets, percent candy offered in the lunchroom, and percent lunchrooms with monitors to help encourage healthy choices. There was an increase of fruit served after the policy implementation and in the percentage of schools stipulating mainly healthy food and beverages for classroom parties. At 1-year, PA opportunities did not change, PE time increased by 14 min/week, recess time decreased by 19 min/week, policies supporting student time in PE and PA engagement during school did not change, PA policy wording was found to be weak. Barriers identified included competing demands, lack of funding and resources, lack of knowledge of the policy, and lack of accountability
Belansky, 2013 [104]	*N* = 10 rural elementary schools were assigned to Adapted Intervention Mapping *n *= 5 or School Health Index *n* = 5; 53% Hispanic (range 20% to 93%); 69% received free or reduced lunch (range 33% to 88%)	The School Environment and Policy Survey to assess changes related to nutrition and PA features of schools were completed by principals, foodservice managers, and PE teachers. Direct observations of schools’ facilities including playgrounds and schools’ buildings features. 37 key informants semi structured interview questions with the Adapted Intervention Mapping schools’ principals, which included debriefing forms and examining the process of achieving behavioral change, facilitators, barriers. 29 key informants interview questions for the School Health Index schools that included logbooks and the status changes of schools resulting of the planning process	Schools		**X**	**X**	Schools using the Adapted Intervention Mapping (AIM) program had 22 total Policy System Environmental (PSE) changes (compared to 3 totals from the other program). Most common change was reversing lunch and recess so that recess came first, making healthy food more available outside of the classroom, and making unhealthy foods less available. PA was increased with 2 schools increasing PE time (e.g., hiring more PE instructors, having smaller class sizes, SPARK curriculum, adding new playground equipment/facilities) and schools implementing changes to the playground and the recess period. Each school made an average of 4.4 effective PSE changes and 90% were maintained one year later
Calancie, 2017 [118]	8 participants of Adam County; 91% White, 2% Black, 6% Hispanic, 1% another race	Interviews on members’ perspectives of the Adams County Food Policy Council Questions included describing the group dynamics, relationships with other council members and partners, activities and participants’ definition of food policy or opportunities for food policy in counties	Local food producers; food retail		**X**		Promoting healthy food access through programs (the ACFPC piloted the Healthy Options program to improve access to fresh, local produce by providing vouchers to participants to use at Adams County Farmers’ Market Association markets); and food policy opportunities and challenges (ACFPC members worked towards policy solutions to address food system issues utilizing programs such as the Healthy Options program)
Carlson, 2017 [169]	2,029 municipalities; urban 1,488 (74.8%) and rural 541 (25.2%); Median education level: high school graduate *n* = 895 (44.4%), college graduate *n* = 1,134 (55.6%); ≥ 20% below poverty level *n *= 614 (30.3%), < 20% below poverty level *n* = 1,415 (69.7%); race/ethnicity: ≤ 50% non-Hispanic white *n* = 269 (13.3%), > 50% non-Hispanic white *n* = 1,760 (86.7%)	National Survey of Community Based Policy and Environmental Supports for Healthy Eating and Active Living and data from the National Complete Streets Coalition database that categorized data at the place, county and metropolitan planning organizations levels to align with local policies	Streets			**X**	11 rural municipalities had Complete Street Policies and 16.8% were not aware of existing policies. Variations in policy reporting between the National Complete Streets Coalition's database and the National Survey of Community‐Based Policy and Environmental Supports for Healthy Eating and Active Living (CBS HEAL) data. As population size increased, prevalence of complete streets policies increased (e.g., from 16.1% in settings with < 2,500 people to 49.6% in those with > 50,000 people) with controls (*p *< 0.001)
Carlton, 2017 [148]	20 public schools, including 13 middle and 7 high schools. 15 schools were rural and the other 5 schools were urban or suburban. All but one school were Title 1 School with a proportion of students who qualify for free or reduced-price lunch program > 40%. Schools had mixed race proportions, including white, Black and Hispanic	Structured Physical Activity Survey to assess PA in schools and the System for Observing Play and Recreation in Communities to assess schools’ facility use and PA settings	Schools			**X**	Shared use/joint use efforts supporting PA included 89% of principles indicated their schools are open to the public after hours, but 87% of the time these schools remain empty, indicating an underutilization of shared use. Perception that schools are off limits may be a barrier to access for PA facilities
Caspi, 2017 [81]	*N* = 732 9th and 10 h grade students; 54% of students were female, 46% were male; 67% were white, 33% non-white; 35% were eligible for free or reduced lunch program and 65% received for full priced lunches	BreakFAST study surveys included questions that addressed factors related to student breakfast patterns, social norms about school breakfast, use of the food environment during the school day, transportation to and from the school, other self-reported health behaviors, and student demographics	Schools		**X**		School breakfast participation was positively associated with fast food purchases for breakfast (OR = 0.979, 95% CI [0.959, 0.999]) but school breakfast participation decreased fast food restaurant purchases on the way home from school (OR = 1.017, 95% CI [1.005, 1.029])
Chrisman, 2014 [149]	407 participants; 57% were female, 43% were men; Education: Less than high school 6.1%, high school 35.1%, some college 32.7%, bachelor’s or higher 26.0%. All were non-Hispanic and white	Questionnaire combining questions from the Behavioral Risk Factor Surveillance System and the National Health Interview Survey on neighborhood characteristics and barriers to being active. The Kaiser Physical Activity Survey asked about domain specific activity levels	Trails; schools; workplace			**X**	PA was positively associated with having a positive attitude toward using government funds for biking trails (*p *< 0.001). The PA summary score across all domains was associated with workplace incentives for exercise (*p *< 0.001) and supporting PE in schools (*p* = 0.047). Regarding the effect between age and support of government funds used to build bike paths, younger adults supported the use of government funds to build biking trails (*p *= 0.023)
Chrisman, 2015 [171]	19 participants, 11 female and 8 males. All were Caucasian	Focus groups interviews to define PA and exercise; neighborhood and community to measure environmental proportions and questions to identify barriers and facilitators of PA in communities	Recreation facilities			**X**	Lack of shared user agreements between facilities and communities was identified as a barrier to PA
Cleary, 2018 [116]	NA	County-year observations for the contiguous U.S. counties’ large food stores to estimate policy outcomes and cost-effectiveness of two types of policies to improve food access in non- metro U.S. areas: 1) food access- demand stimulating policies, such as increase in Supplemental Nutrition Assistance Program dollars, and; 2) supply-side policies, such as subsided to reduce establishment costs; by estimating the minimum market size needed for more large stores in non-metro U.S. countries to be profitable	Food retail		**X**		Neither type of intervention is preferred a priori, and that the cost-effectiveness of each policy type depends upon the presence of an adjacent metropolitan county, the number of pre-existing stores, and the duration of the demand-side stimuli. Results suggested that the cost-effectiveness of broad-based policy solutions to improve physical access to large food stores or to stimulate demand may be limited when it comes to easing entry in areas with multiple stores
Demment, 2015 [82]	17 middle schools; 53% male and 47% female; 43% were low income at birth and 57% were not low income at birth; 96% white, 43% of students were < 185% of the poverty line	The Nutrition and Physical Activity environment assessment tool based on the School Health Policies and Programs Study questionnaire and the Eat Well Be Active questionnaire	Schools		**X**	**X**	Study found schools could not be broken down simply into "health promoting" or "not health promoting". Each school had differing needs, and the recommendation was for an initial school environment assessment to be made before schools begin forming health promotion policies to be the most effective. Food fund-raising policies received the most variation score but school meal quality/availability received the least variation score
Drummond, 2009 [123]	17 practices in Yuma County; Hispanic: 55.5%, < poverty level: 17.6%	The Nutrition and PA Self-Assessment for Child Care included workshops to raise awareness of childhood obesity, and self-assessments questionnaire adopting 56 best practices in nutrition and PA	Childcare		**X**	**X**	After a childcare staff and administrator intervention (worships, self-assessments, action plans, and technical assistance), the majority of improvements made were regarding policies for menu and snack varieties. Some centers changed PA policy as well to include more outside time and walking "mini field trips" during the day
Dumphy, 2016 [69]	Pre-implementation group *(n* = 43) healthy newborns and post-implementation group (*n* = 45) healthy newborns	Electronic medical records were used to collect a pre-implementation and a post-implementation data during each routine newborn, 1-month, 2-month, and 4-month well-child visit	Workplace	**X**			After implementing the Academy of Breast-Feeding Medicine protocol policy to improve workplace support, breast feeding rates for any breast feeding increased post-implementation. There was a positive relationship between implementing a breast-feeding friendly office protocol and breast-feeding rates over time
Evenson, 2011 [158]	183 municipalities, 108 < 5,000 (rural) and 75 ≥ 5,000 (urban)	Survey targeted at the North Carolina municipal staff member most knowledgeable and asked about walking and bicycling projects, programs and policies	Trails; streets			**X**	Plans, projects, programs, and policies were all more likely to exist in urban places vs rural. Municipalities had policies related to maintaining sidewalks, trails, footpaths and crosswalks (63.6%), building sidewalks, trails and greenways (57.2%), restricting the speed or access of automobiles (45.2%), and enhancing pedestrian facilities in new developments (43.5%)
Farris, 2014 [92]	3 schools in Montgomery County with 87.9% white, 4.1% Black, 2.9% Hispanic/Latino; Giles County with 97% white, 1.5% Black, 1.3% Hispanic/Latino	Observational protocol carried out by undergrad and grad nutrition students for visual item identification and portion size estimation	Schools		**X**		School lunches, observational data from 3 schools in rural Virginia for 5 consecutive school days findings were that school lunches more likely to meet nutritional standards than packed lunches, especially for fat and saturated fat levels. National School Lunch Program (NSLP) participants consumed fewer solid fats and added sugars than packed lunches. Packed lunches were less likely to contain fruits (54% vs 67%), vegetables (17% vs 61%), juice with no sugar (10% vs 22%), and milk (20% vs 96%) than NSLP meals. They also contained more savory snacks such as chips and crackers (57% vs 5%) and sugar sweetened beverages (40% vs 0%)
Findholt, 2011 [94]	Six high school students, one from each community in Union County; 4 female, 2 male; all white	Photovoice was used to engage youth in the community assessment, obtain their perspective of assists and barriers and build support for future intervention by raising awareness of community conditions affecting diet and PA	Schools		**X**	**X**	A major barrier to PA was the limited recreational facilities outdoors. Street safety concerns was another reported barrier to PA. Schools did not offer enough PE and recess classes. Proximity to natural resources and youth sports increased PA level in children. Access to healthy eating was expensive and limited; whereas, fast food options were convenient and readily available
Flower, 2008 [29]	1,292 women from North Carolina and Pennsylvania; low-income in NC (*n* = 168) and not low-income (*n* = 86) participants, low-income in PA (*n* = 344) and not low-income (*n* = 175) participants. Mother has high school degree/GED/ 83%, Mother has college degree (4 years)/ 20%, Mother employed/ 41% African Americans in NC (*n *= 521) and Others predominantly Caucasian, predominantly Caucasian PA sample	Ethnographic interviews combining quantitative and qualitative data from Family Life Project and demographic information including maternal and infant health factors and health services to predict breastfeeding initiation and continuation	Food assistance programs	**X**			WIC supply of formula encouraged one woman to stop BF. Mothers also shared the perceptions that WIC staff were not consistent about promoting breast or formula feeding. WIC participation was associated with not BF
Foster, 2015 [124]	29 childcare centers, all within low-income rural communities, locations: Indiana *n* = 2, Kansas *n* = 4, Michigan* n* = 2, North Dakota *n *= 2, Ohio *n* = 8, South Dakota *n* = 9, and Wisconsin *n* = 2	The Young Men Christian Association's Community Healthy Living Index Early Childhood Program Assessment Tool to assess early childcare centers’ physical environment, promotional efforts and polices related to nutrition and PA that include questions on "healthy eating opportunities" and "general healthy living," and assessing respondents’ confidence	Childcare		**X**	**X**	Three states (Michigan, Ohio, and Wisconsin) had nutrition policies in line with Healthy People 2020, and 3 more (Indiana, Kansas, and South Dakota) had less stringent policies. Policies were not consistently enforced, however. All states but North Dakota had policies regarding gross motor movement, but not moderate to vigorous PA in childcare centers. Centers indicated a lack of funding and staff training as a barrier to enforcing PA and nutrition policies
Friedman, 2014 [119]	13 providers Patients were 18.2% male and 81.8% female; 31.2% less than high school, 40.9% high school graduate or GED, 15.9% some college or technical school, 11.4% college graduate or graduate school; 93.2% African American and 6.8% white	Provider Surveys questions included *“how many prescriptions did you give out? Did you target your prescriptions to any specific group? If yes, which groups? How did your patients respond to the prescription program? Did you typically give the prescription in conjunction with other prescriptions? How did you typically present the prescription to your patients? In general, how easy was it to give out the prescription? What made it easier for you to use the prescription? What made it difficult for you to use? the prescription? (options provided); What could be done to improve the prescription program? How important is it that there is a farmers’ market at FHC in the future? How much do you agree with the following statement—The farmers’ market provided important health benefits to PATIENTS at FHC and How much do you agree with the following statement— The farmers’ market provided important health benefits to STAFF at FHC How often did you shop at the farmers’ market?”*	Healthcare; food retail		**X**		Outcomes from a Farmer’s market prescription and voucher program showed most physicians *(n *= 5; 55.6%) reported distributing 25 or more prescriptions to patients. Two distributed less than five and two did not give prescriptions
Friesen, 2010 [95]	*N* = 1,666 participants; *n* = 507 (31%) male, *n* = 1,130 (69%) female; *n* = 961 individuals enrolled in at least one Wellness University fitness class; 80.5% (*n* = 775) female and 19.5% (*n *= 187) male	The PA Readiness Questionnaire to measure changes in nutrition, and PA related to knowledge, attitude, and behaviors by completing a pre/post assessment and report “stage of change” scores on 8 health related behaviors, including “ability to improve the types of healthy foods I eat, lose weight, eat 2–3 servings of fruit every day, eat 2–3 servings of vegetables every day, eat whole grain products every day; eat nonfat dairy products every day, get 30 min of aerobic exercise five times per week; and, if a smoker, to quit smoking”	County fair; parks; schools; workplace		**X**	**X**	Participants of the Operation Wellness intervention which utilized two components to target different populations (Wellness Preparatory for toddlers through middle school aged children and Wellness University for high school students and adults) engaged in PA on significantly more days per week over the 4 years of the intervention. One example of a cost-effective, successful wellness university program was the Dump Your Plump Worksite weight loss competition, where 333 individuals on 52 different worker-led teams lost over 1ton (*n* = 2,256 pounds) of body weight. This program also lobbied and successfully implemented policies which added vended milk machines in schools, removed deep fat fryers, and implemented healthy food policies for meetings at workplaces and schools, banned donuts at police departments, and added a centralized walking path on the county fairgrounds. 11.5% of respondents reported consuming the recommended amount of fruit and vegetable daily; no significant difference in consumption servings of produce was reported (*p* = 0.98). 22.9% consumed the recommended dairy products daily with no significant differences reported in consuming dairy product (*p* = 0.91)
Gamble, 2017 [150]	4 total focus group discussions; 2 with principals and PE teachers; 2 with students in 4th and 5th grades	Semi-structured focus groups discussed in-school PA opportunities, barriers, facilitators to implementing in schools PA	Schools			**X**	Support by school administration was identified as an enabling factor for implementation of in school PA policies. Other positive factors included recess, classroom integrated PA, school wide breaks, and scheduled PE classes. Support from policymakers was also needed as there is limited or no support provided to the Mississippi Healthy Students Act implementation
Hafoka, 2017 [142]	3 towns: Population in town 1* N* = 3,292, 87.4% (*n* = 2,285) high school graduates or higher, 59.5% (*n* = 1,960) Native Hawaiian and Other Pacific Islander. Population in town 2 *N* = 6,419, 97.9% (*n *= 6,009) high school graduates or higher, 56.8% (*n* = 3,292) Native Hawaiian and Other Pacific Islander. Population in town 3 *N* = 5,555, 87.4% (*n *= 3,625) high school graduates or higher, 70.2% (*n *= 3,904) Native Hawaiian and Other Pacific Islander	Rural Active Living Assessment, The Street Segment Assessment, The Town Wide Assessment, and The Policy and Program Assessment tool has 20 items to identify programs and policies in towns and schools that promote PA	Schools; streets			**X**	All rural towns in Hawaii, (*n *= 3) scored a 0 on the town policies portion of the policy and program assessment, meaning there were no PA promoting policies in the town as a whole. Important to note that the PA only has one item to identify town policy. Overall mean score was 39 out of 100 for all three towns. Average score of 10 out of 30 for school policies and 15 for school programs. Town 2 had the most school policies and programs, which may be linked to their higher availability of sidewalks, crosswalks, and existence of a bike lane
Hill, 2016 [170]	Total sampled residents *n *= 813, 23.9% male and 76.1% female; Less than high school = 17.4%, High school or GED = 36%, 1–3 y college = 30.8%, ≥ 4 y college = 15.9%; white: 61.7%, Black: 35.5%, Other and multiracial: 2.8%	Behavioral Risk Factor Surveillance Survey data, the Physical Activity Resource Assessment, The Neighborhood Environment Walkability Survey, and the Godin-Shephard Leisure Time Exercise Questionnaire measures PA duration within the next 7 days	Recreation facilities			**X**	This study identified PA outlets in rural areas. The study lists four strategies to encourage PA despite the identified environmental barriers. These strategies include shared-use agreements. There were significantly more pay-for-use outlets within urban areas (*p* < 0.05). Rural PA outlets had a significant higher count of features (*p *< 0.05) than urban outlets. Incivilities “litter and issues with grass" in rural outlets were significantly highly rated than in urban outlets (*p *< 0.05)
Horne, 2013 [130]	Schools, worksites, Community at large (Klickitat County), health care facilities, community institutions or organizations; total 23 areas	A telephone interview to assess leaders’ challenges regarding implementing the Action Communities for Health, Innovation, and Environmental Change model through The Healthy People Alliance that used the CDC’s Community Health assessment and Group Evaluation tool	Community gardens; trails; workplace		**X**	**X**	Difficult to implement the Action Communities for Health, Innovation, and Environmental Change ACHIEVE model (addresses multiple chronic disease factors through policy, systems, and environmental approaches) due to lack of availability of coalition members to be involved in community efforts. Need for additional funding was also a challenge. This model successfully established community gardens, enhanced a walking and biking trail, and developed and promoted a local worksite wellness toolkit
Izumi, 2006 [83]	383 directors; average free or reduced lunch participation rate is 36% for 2002–2003	The Michigan Food Service Director Farm-to-School Survey adapted from the Oklahoma Survey of Institutional Food Service Providers that assessed schools’ food service directors’ interest in implementing a farm to school purchases, practices preferences, concerns, and barriers	Local food producers; schools		**X**		Average free and reduced lunch participation rate was 36%, compared with the statewide rate of 39% for the 2002–2003 school year. Seventy-three percent of food service directors expressed interest in purchasing food directly from a local farmer if price and quality were competitive and if a source was available. More than 10% of respondents indicated a willingness to pay higher prices for locally grown foods. Close to half (45.5%) of food service directors reported that their institutions would not be willing to pay a higher price to purchase local foods, while 43% of respondents were neutral. The most frequently cited concerns about purchasing locally produced foods were cost (76.2%), reliable supply (71%), seasonality of fruits and vegetables (68.7%), food safety (66.8%), delivery considerations (60.8%), and quality (52%). Food service directors also reported a number of barriers that would prevent purchasing foods directly from local producers including federal and state procurement regulations (71%), lack of products available during certain times of the year (64.2%), and lack of local producers in area from whom to purchase (52.7%)
Jantzer, 2018 [70]	*N *= 135 individuals (*n *= 125 women, *n* = 12 men); 98.9% Caucasian, *n *= 1 Native Hawaiian or Pacific Islander; 90% a bachelor’s degree or higher, 37% graduate degrees	Greene and Olson’s (2008) Employee Perceptions of Breastfeeding Support Questionnaire to measure breastfeeding support in the workplace included questions on company policies/work culture, manager support, coworker support and workflow	Workplace	**X**			As workplace support for BF increased, particularly when employees have enough time to express human milk at work, employees perceived greater work enhancement of their personal lives. Conversely, as workplace support diminished, especially in terms of lack of time to express human milk, employees perceived greater work interference with personal life
Jernigan, 2012 [128]	40 focus group participants	Tool for Health and Resilience in Vulnerable Environments (THRIVE) to assess social and environmental factors that affect health in communities. Questions include *“What do you see here? What’s really happening here? How does this relate to our lives? Why does this problem, concern or strength exist? and What can we do about it?”*	Local food producers		**X**		Coalition members established a Producer’s Guild to connect agricultural producers to community coalition members, as well as other key stakeholders, to develop a Community Supported Agriculture program
Jilcott Pitts, 2013 [115]	Lenoir population: 59,495 persons; poverty rate 23.2%; 51% white, 40% Black. The Heart Healthy Lenoir intervention population: 366 76.0% female; and 65.6% Black, 33.3% white, and 1.1% other race	Group discussion with Community Advisory Council members (*n *= 19), in-depth interviews with stakeholders (*n *= 11), and a quantitative survey with the Heart Healthy Lenoir intervention participants (*n *= 366) to rate winnable strategies and policies’ recommendations based on culture, infrastructure, community leader support, and funding	Food retail; trails		**X**	**X**	The Common Community Strategies for Obesity Prevention (COCOMO) is a set of 24 CDC recommended community-level obesity prevention strategies. The Community Advisor Council found that the least frequently selected COCOMO strategy was limiting advertisement of unhealthy food and beverages and the most frequently selected strategy was the increase of opportunities for PA within the community. Reasons for why limiting advertisements was the least selected include that it would be difficult to monitor, would interfere with business marketing, presence of existing healthy options at restaurants and that individuals would be uncomfortable with the government telling them what to eat. Barriers for increase in physical opportunities included lack of funding for expensive infrastructure, such as bike lanes, and perceived lack of safety in less policed areas. Funding was identified as a barrier to implementation of PA opportunities and a facilitator for policy and environmental changes
Jilcott Pitts, 2015 [131]	(*N *= 366) participants, *n *= 278 (76%) females, mean education 13 years, *n *= 236 (65%) Black, *n *= 128 (35%) non-Black	Community audits and surveys on perceptions of neighborhood barriers that included questions about factors that hindered PA and healthy eating	Neighborhoods		**X**	**X**	Those that perceived more nutrition barriers in their neighborhoods had higher support for policy change. Those who had more PA resources around their residences had higher support for PA policy changes
LaJeunesse, 2019 [143]	Nonparticipating schools (*n *= 1,749), schools’ income level: high (Low = 16.2%), (Medium = 62.9%), (High = 20.9%), 14.1% Hispanic, 23.8% Black, 51.2% white, 3.7% two races; Participating schools (*n *= 853), schools’ income level: (low = 19.6%), (medium = 55.5%), (high = 24.8%), 14.8% Hispanic, 25.1% Black, 50.0% white, 3.8% two races	County level data obtained from the North Carolina Center for Public Policy Research, and school level data was obtained from the National Center for Education Statistics, the North Carolina Department of Public Instruction	Schools; streets			**X**	Rural schools (*n *= 373) were less likely to participate in Walk to School days than suburban (*n *= 186) and urban (*n *= 291) schools but were as likely to institute Bike to School days and recurring bike-walk programs. Rural schools had an odds ratio of 0.555 odds of having a Walk to School Day, results indicating that lower income schools were more likely to establish recurring bike walk programs than their wealthier counterparts
Lange, 2019 [117]	27.3% Rural (≤ 50% urban), 72.8% Urban (> 50% urban), 44% ≤ High school, 56.0% had a median education level of some college or higher, 86.9% > 50% non-Hispanic white, 13.1% ≤ 50% non-Hispanic white	The National Survey of Community-Based Policy and Environmental Supports for Healthy Eating and Active Living	Food retail		**X**		Municipalities with ≥ 50,000 people were significantly more likely to incentivize farmers markets, supermarkets, convenience and corner stores compared to municipalities with < 2,500 people. Incentives were commonly used in Midwestern supermarkets and least likely to be used in Western supermarkets (*p *< 0.001). Convenience and corner stores used incentives twice likely in municipalities where ≤ 50% of the population non-Hispanic white (*p *< 0.001)
Larson, 2018 [84]	16 schools (8 Full intensity intervention and 8 Grab and Go component); 364 students enrolled at Time 1; 47% female, 9th and 10th grades, *n *= 126 eligible for free/ reduced price school meal and *n *= 238 not eligible for free/ reduced price school meals; 83% (*n *= 284) non-Hispanic white, Hispanic or non-white 17% (*n *= 80). One school in a city, 4 schools in communities classified as rural town fringe and 3 schools in rural communities classified as small towns. Seven schools implemented Grab and Go during Time 3, which included 55% female; 35% eligible for free/ reduced school meal; 78% non-Hispanic white	Evaluation of grab-and-go style breakfast carts and policies that allowed students to eat outside the cafeteria. School record data collected at baseline, during, and after the Project Breakfast intervention implementation. Collected data include the number of attendance days and the number of days school breakfast was purchased	Schools		**X**		School Breakfast Program participation increased from baseline for schools enrolled in the grab-and-go only component (13.0% to 22.6%). Student increases in school Breakfast Project participation were observed among the at-risk sample (7.6% to 21.9%) and among subgroups defined by free-or reduced-price meal eligibility and ethnic or racial background. Participation in School Breakfast Program increased among students eligible for free or reduced-price meals from 13.9% to 30.7% and among ineligible students from 4.3% to 17.2%
Liberty, 2019 [61]	*N *= 10,343 births at a Baby Friendly Hospital; maternal residence at a metropolitan area: 8,937 (86.4%), at non-metropolitan area 1,406 (13.6%)	Baby Friendly Hospital designation status was assessed by publicly available data from Baby-Friendly USA and statewide geospatial data; The primary outcome/ breast feeding initiation	Hospitals	**X**			Giving birth at a Breastfeeding Friendly Hospital (BFH) increased the odds OR = 1.88, 95% confidence interval [1.77, 2.00], of initiating BF when compared to giving birth at a non-BFH. Birth at a BFH predicted breastfeeding initiation OR = 1.82, 95% confidence interval [1.65, 2.01]. Maternal residence OR = 0.81 (0.72, 0.92) and hospital exposure both increased the probability of initiating breastfeeding for rural counties of > 20,000 residents, 2,500 residents, and < 2,500 residents
Lillehoj, 2012 [62]	*N *= 53 hospitals, 11.3% governmental, *n *= 6 (11.3%) teaching, *n *= 9 (17%) private, *n *= 42 (79.2%) general hospitals	Hospital Breastfeeding Policies: Self-Appraisal Pre-Assessment, a WHO/UNICEF assessment using stakeholder discussion by visiting a list of hospitals to explore current policies related to breastfeeding and maternity and introduce the Baby‐Friendly USA and the Breast-Feeding Hospital Initiative Ten Steps and know interest to attain designation status; in addition to reviewing the state and individual breastfeeding rates and opportunities for hospital level improvement based on the Hospital Breastfeeding Policies: Self‐Appraisal Pre‐Assessment survey participation	Hospitals	**X**			Of hospitals assessed (*n *= 65), *n *= 55 (81%) were in rural Iowa counties. Of the ten steps of Baby Friendly Hospital Initiative (BHHI), Proportionally, rural hospitals were less likely than urban hospitals to have implemented step 10 (policy implementation for provision of community resources and referral information related to breastfeeding; *p *= 0.01); no other statistically significant differences between urban and rural hospitals were identified. Overall, rural hospitals were on par with urban hospitals regarding adopting Baby-Friendly initiatives, aside from step 10. The most widely adopted policy, encouraging breastfeeding on demand, was implemented by 83% of the hospitals
Lillehoj, 2016 [161]	26 counties were assessed;14.4% poverty rate; high school graduation: 89.1%, 89.8% white	Community Health Assessment and Group Evaluation (CHANGE) tool to assess the document policy and environmental changes in communities	Sidewalks; streets; parks; workplace			**X**	In assessment of environmental community aspects, Complete Streets, bicycle use, and street calming were found to need improvement. Land use plans, maintain parks, and sidewalks compliant with the Americans with Disabilities Act were identified as strengths. Worksites scored low on promotion of stairwells, encouraging non-motorized commuting, and implementing activity breaks but scored high on subsidizing gym membership and providing area for PA
Lo, 2017 [140]	Median Household income below $50,000; all were non-Hispanic white	Inventories for Community Health Assessment in Rural Towns, which assess active living characteristics related to PA	Recreation facilities; streets; schools			**X**	Eight built environment audits, 15 resident focus groups (118 adults), and 24 key informants' interviews were conducted between August and December 2014. Residents cited poor city planning and lack of maintenance as barriers to built environments to aid in PA. In all focus groups, participants described the presence of PA facilities, such as sports fields, recreation centers, swimming pools, or gyms as well as non-traditional or mixed-use spaces like school athletics facilities. Key informants criticized the lack of sidewalks and sidewalk quality in one community, the key informants reported improvements in these features, suggesting differences in political agenda and priorities between jurisdictions
Majee, 2016 [71]	*N *= 17 mothers; six had a college education, 17.2% of the community live in poverty; *n *= 3 Hispanic white, *n *= 14 white- non-Hispanic Americans	Semi structured interviews and focus group questions with mothers about workplace supports for breastfeeding and breastfeeding while at work experiences	Workplace	**X**			Workplace barriers to breastfeeding included the extent that workplace policies were flexible (e.g., break frequency and time to support pumping), intolerant (e.g., being taunted at work for taking breaks to breastfeed), and lack of proactiveness by employers (e.g., encouraging the practice of breastfeeding)
Mann, 2017 [85]	7 rural middle schools; grades k to 8; mean school compliance: 10.3 (SD = 6.8); average free/ reduced lunch eligibility: 57% (range 51.0%-63.0%); 93% of students white	Beverage and Snack Questionnaire 2 with the addition of flavored milk, water, and coffee or tea, designed for children aged 10–18 years	Schools		**X**		School nutrition standards were effective at increasing compliance for a la carte items (*p *< 0.001) and beverages (*p *= 0.009). In addition, more foods met calorie, fat, and sodium standards after implementation of the new nutrition standards
Mayo, 2013 [99]	Region 9 includes 15 counties (Bertie, Camden, Chowan, Currituck, Dare, Edgecombe, Gates, Hertford, Hyde, Martin, Northampton, Pasquotank, Perquimans, Tyrell, and Washington); All regions considered rural except two (Currituck and Edgecombe); 77.9% of residents live in rural areas; 37% African American	Policy coding forms to examine “the relationship between the built environment and health and local government laws and policies and the food code/policy audit form to code and score the region’s 9 counties and municipalities in 4 areas. Interviews between county’ planning directors and municipality’ staff to discuss the “implementation and enforcement of zoning ordinances.”	Food retail		**X**		Healthful food zoning score was positively correlated with the number of fruit and vegetables outlets with a low mean of 0.33 of 1 (range: 0–0.83) in 13 counties. Major themes in implementation and enforcement of zoning to support fruit and vegetable outlets included strict enforcement (e.g. not allowing the operation of a fruit or vegetable outlet on land not zoned for such purposes) versus lack of enforcement of zoning regulations (e.g. an area zoned as rural residential has ability to host farmers markets and produce stands, but other properties that aren’t zoned for this aren’t regulated)
Meendering, 2016 [96]	151 public schools	The Wellness School Assessment Tool to assess schools’ federal requirements through the comprehensiveness score and the language used within the policy through a strength score	Schools		**X**		Significant differences were observed between large school districts’ comprehensiveness (*p *= 0.009) of wellness policies compared to small and medium school districts. Large school districts also have lower policy strength (*p *= 0.035) than small schools. Total comprehensiveness scores of large schools were significantly lower than small schools. Total strength score of larger schools was lower than medium and small schools
Merewood, 2005 [64]	29 hospitals; 9 rural hospitals; 8 were community hospitals, 1 was a Free-standing birth center	Baby-Friendly USA provided a list of U.S. hospitals meeting the status across the U.S. in 2001 (*N *= 29). The Baby-Friendly coordinator was phone interviewed and asked about meeting Baby-Friendly status criteria. Obtained data used to assess the association between hospital and patient characteristics and rates of newborn breastfeeding initiation and exclusivity	Hospitals	**X**			All 9 Hospitals provided breastfeeding initiation rates. The mean breastfeeding initiation rate for the 9 Baby-Friendly hospitals in ranged from 98–65%. Four out of nine rural hospitals did not provide data on exclusive breastfeeding and five rural hospitals provided data on exclusive breastfeeding
Metos, 2007 [93]	30 schools; (*n *= 14) 50% were rural; (*n* = 1) 3.33% < 50% Caucasian, (*n *= 2) 6.66% 50–75% white, (*n *= 27) 90% 75–100% Caucasian	The Strength of School Wellness Policies (SOSW) to assess national and local recommendations on local school wellness polices in response to Child Nutrition Reauthorization Act (CNRA) collected using phone call and websites	Schools		**X**	**X**	The majority of all Utah school districts (78%) complied with federal guidelines, including a variety of state recommended nutrition and PA policy statements. Districts were more likely to mandate items already required by other entities. School districts with high participation in free and reduced-price programs had significantly more mandatory policies (mean = 9.2) versus low (mean = 7.1) and medium enrollment (mean = 4.7). The mean of mandatory policy language related to competitive foods in rural districts was 0.93 (SD = 1.13) in 14 schools. The mean of mandatory policy language related to PA in rural districts was 2.62 (SD = 1.55) in 13 schools
Moore, 2010 [151]	Seven rural areas; 41 parents (20 rural); 10% male and 50 students (22 rural); 44% male; 58% 6th grade. 19.6% of parents had less than 9th grade, 9.8% had some high school, 9.8% had a high school diploma or equivalent, 11.8% had some college, 15.7% had an associate degree, 15.7% bachelor’s degree and 7.8% a graduate or professional degree; 19.6% of parents Hispanic or Latino, 21.6% considered child as Hispanic or Latino	Focus groups questions related to the socio-ecologic framework and PA	Schools			**X**	Child Perceived Barriers to PA, which included school PA Policies (8th graders do not get recess time), lack of culturally appropriate facilities, and programming, cost of recreational activities e.g., baseball membership, danger of crimes in neighborhoods, parental modeling of PA, sedentary behaviors and TV watching
Moore, 2017 [113]	400 governments	38 item survey that inquired about policies and standards local governments can implement for PA and healthy eating as well as, respondents’ experiences in completing the survey with the local government. There were 3 questions on community wide planning, including whether the local government implemented a comprehensive/ general plan, a master plan, or selected nutrition and PA objectives in planning documents	Food assistance programs; food retail; recreation facilities; streets; parks		**X**	**X**	For populations of < 1,000 (*n *= 45): 38% had policies that support bike/pedestrian friendly design, 8% had policies that support complete streets, 0% had policies that support bicycle racks required at public facilities, 34% had policies that support pedestrian friendly policies for new or retrofit development, 83% had policies or budget provisions to support activity in parks and rec areas, 23% had policies that support joint use agreement to allow public use of school recreational facilities; 0% had nutrition standards in government buildings or worksites, 13% had policies to support transportation to healthier food retailers, 0% had policies that support funding for EBT in farmers markets, 5% had food policy council, and 36% had breastfeeding friendly policies. For populations of 1,000–4,999 (*n *= 53): 41% had policies that support bike/pedestrian friendly design, 12% had policies that support complete streets, 11% had policies that support bicycle racks required at public facilities, 65% had policies that support pedestrian friendly policies for new or retrofit development, 85% had policies or budget provisions that to support activity in parks and rec areas, 43% had policies that support joint use agreement to allow public use of school recreational facilities; 2% had nutrition standards in government buildings or worksites, 28% had policies to support transportation to healthier food retailers, 4% had policies that support funding for EBT in farmers markets, 6% had food policy council, and 39% had breastfeeding friendly policies. For populations of 5,000–24,999 (*n *= 48): 64% had policies that support bike/pedestrian friendly design, 15% had policies that support complete streets, 41% had policies that support bicycle racks required at public facilities, 82% had policies that support pedestrian friendly policies for new or retrofit development, 87% had policies or budget provisions that to support activity in parks and rec areas, 65% had policies that support joint use agreement to allow public use of school recreational facilities; 0% had nutrition standards in government buildings or worksites, 38% had policies to support transportation to healthier food retailers, 0% had policies that support funding for electronic benefit transfer in farmers markets, 7% had food policy council, and 70% had breastfeeding friendly policies
Morshed, 2015 [97]	Fall 2008: *n *= 74, 48.7% female, 4.2% 8th grade or less, 9.9% some high school, 63.4% high school graduate or GED, 14.1% some college, 7% associate degree, 1.4% bachelor’s degree or higher; 56.8% Hispanic, 43.2% non-Hispanic, 59.5% White, 32.4% American Indian, 2.7% Asian, 5.4% mixed race. Spring 2010: *n *= 88, 50% female; 4% 8th grade or less, 17% some high school, 38% high school graduate or GED, 18% some college, 7% associate degree, 2% bachelor’s degree or higher; 53.4% Hispanic, 46.6% non-Hispanic, 48.9% white, 44.3% American Indian, 6.8% mixed race	Proxy interviews with caregivers about the home environment, dietary intake and behavior, PA, and family demographics. Modified version of the Block Kids 2004 Hispanic Food Frequency Questionnaire (FFQ) to assess food intake in last week, included questions about the amount of food the child consumed each day and on how many days in the previous week the food was consumed. It also identified foods or food categories typically consumed by the study population	Schools; food assistance program		**X**		The consumption of lower fat milk was significantly increased; there was reduced consumption of saturated fat (from 22.31 g to 19.99 g on average), and decreased consumption of vegetables without potatoes (from .80 cups to .66 cups on average) were reported following policy change. There were no observed significant differences in fruit, fruit juice, vegetables including potatoes, whole grains, and saturated fat consumption
Mucioki, 2018 [109]	41.17% of survey respondents used at least one type of other food assistance, and 36.23% did not use food assistance, 60% relied on food assistance program	Key informant interviews, focus groups, and a household survey that were co-created with tribal partners to assess food access and food systems	Food assistance programs; food retail; local food producers		**X**		Interviews and focus groups with tribal members reported that the Federal Distribution Program for Indian Reservations (FDPIR) was essential to food security especially for children; all focus groups noted food assistance programs such as FDPIR to be extremely important. Some users reported that FDPIR helped alleviate financial stress by reducing expenses related to grocery shopping. Many participants reported improvement in the food quality (including the addition of fresh fruits and vegetables) that were made possible through community advocacy. Participants also noted the importance of transitioning from nationally sourced goods, to local sources of produce that may be more culturally relevant and accessible by the community
Nanney, 2019 [86]	16 rural schools (8 intervention and 8 control schools); 48.2% in 10th grade and 51.8% 9th grade; 32.2% the median percent of students eligible for free/ reduced price meals; 87.8% median percent of students' non-Hispanic white	The Project breakFAST intervention was used to address environmental and social factors that motivate students to eat a school breakfast. Data about students who received a fully reimbursable school breakfast, the number of school days students’ attend and the mean School Breakfast program participation rate was obtained through the company the school used for data management	Schools		**X**		An intervention focused on increasing student access to breakfast by changing school services practices and by promoting school breakfast through student-directed marketing campaigns was implemented over 12 months across 9th-10th grade students in 16 rural high schools in Minnesota. School Breakfast Program (SBP) participation rates increased from baseline to follow-up by a median of 3% for the intervention group and intervention schools
Ndirangu, 2007 [110]	21 community members and nine university representatives	Conducted a needs assessment using the Comprehensive Participatory Planning and Evaluation model to assess nutrition and health problems; a workshop to develop a menu of interventions	Food assistance programs; neighborhoods		**X**	**X**	Community members and partners utilized the CPPE model to identify problems and barriers to nutrition and PA. Problems identified included unhealthy foods and lack of adequate PA. Restrictions that govern the use of food stamps as well as the misuse of food stamps were identified as barriers to healthy eating for residents
Omura, 2017 [152]	1,930 municipalities; 511 in rural areas; Median education level: 854 high school graduate or lower, 1,076 some college or higher; Race/ ethnicity: 258 ≤ 50% non- Hispanic white, 1,672 > 50% non- Hispanic white	National Survey of Community Based Policy and Environmental Supports for Healthy Eating and Active Living with questions about shared use agreements	Schools			**X**	City and town managers reported 28.9% of rural areas had shared use agreements and reported the highest proportion of shared use agreements with outdoor spaces (~ 58%), followed by outdoor and indoor space, and lastly indoor spaces exclusively
Onufrak, 2016 [133]	1,945 municipalities; *n *= 522 rural	Used data from the National Survey of Community-Based Policy and Environmental Supports for Healthy Eating and Active Living survey of 1,945 municipal governments serving populations of 1,000 or more to assess the presence of written nutrition standards, food groups or nutrients addressed by standards and the population served by facilities	Local government; workplace		**X**		Rural communities (*n *= 522) reporting written nutrition standards for foods sold or served in local government buildings or worksites among municipalities was 2.1% (95% CI: 0.9–3.4%)
Park, 2018 [134]	*N* = 2,029 municipalities; *n *= 541 rural (25.2%); poverty prevalence: 69.7% municipalities < 20%, 30.3% municipalities ≥ 20%; median educational attainment: 44.4% ≤ High school graduate, 55.6% ≥ some college; racial ethnic composition: 49.9% of municipalities were 51% to 89% non-Hispanic white	The Community-Based Policy and Environmental Supports for Healthy Eating and Active Living survey	Recreational facilities; parks			**X**	Municipalities with population size of 2,500–49,999 people had higher odds of having written plans for providing free drinking water in outdoor public places (adjusted OR, 1.75; 95% CI, 1.31–2.34) or those with population size of ≥ 50,000 people (adjusted OR, 2.52; 95% CI, 1.51–4.22) compared with those with 1,000–2,499 people. Municipalities with 2,500–49,999 people had higher odds of having policies or budget provisions for free drinking water in parks/outdoor recreation areas (adjusted OR, 1.80; 95% CI, 1.34–2.40) or those with population size of ≥ 50,000 people (adjusted OR, 4.17; 95% CI, 2.38–7.29) compared with those with 1,000–2,499 people
Perry, 2015 [139]	Town 1 population *N *= 3,227 (44%), $38,400 median household income, 89.9% (± 3.8) Hispanic; Town 2 population *N *= 10,893 (73%), $39,709 household income, 74.0% (± 4.1) Hispanic; Town 3 population *N *= 15,940 (67%), $34,698 median household income, 80.7% (± 3.0) Hispanic; Town 4 population *N *= 8,970 (30%), $29,692 median household income, 80.4% (± 4.5) Hispanic	Rural Active Living Assessment	Schools; streets			**X**	For program and policy assessment town policies received an overall mean of 5.8 (SD = 5.1) out of 10, town programs received a score of 19.5 (SD = 13.4) out of 30, school policies 22.5 (SD = 8.7) out of 30, and school programs received a score of 21.3 (SD = 7.5) out of 30. Total policy assessment score was 69 out of 100 overall. Two towns did not offer sliding fees scales for their programs, creating a barrier for low-income citizens. Two towns had a policy requiring bikeways and walkways for new infrastructure. All four school districts allowed public access to schools after school hours. All four school districts offered school programming, but only two offered late bus options
Peterson, 2018 [159]	*N *= 1,786 municipalities; 60.6% rural (≤ 5 0% urban); 69.5% High school graduate and 84% college graduate; 83.9% ≤ 50% non-Hispanic white, 77.1% > 50% non-Hispanic white	The study assessed the prevalence of reported plans among U.S. municipalities by municipality characteristics using the National Survey of Community-Based Policy and Environmental Supports for HE and Active Living	Sidewalks; streets; trails			**X**	89% of the municipalities had a comprehensive plan, a bicycle or pedestrian plan, or a land use plan. 64% had a comprehensive/general plan, 46% had a transportation plan, 48% had a bicycle or pedestrian plan, and 76% had a land use plan. 78% of municipalities with a plan included at least one of the three objectives measured supportive of active living
Riley-Jacome, 2010 [153]	17 participants (15 females, 2 males); 52% attended some college; 42.5% retired	Questions from the Behavioral Risk Factor Surveillance System modules on walking, leisure time PA and previous exercise history. Focus Groups with school administrators to select school location and then focus groups with program participants	Schools			**X**	Building facilities, distance to school buildings, conflicts with other school activities, and lack of personnel to administer the program were identified by all three groups as barriers to implementing and utilizing a school walking program. Site specific barriers: building temp, hours of operation, school closings. Personal barriers included caretaker duties, time constraints, boredom. Administrators were uniformly very supportive of their walking program and recommended that other districts implement similar programs. Using school facilities can be an inexpensive and effective means of increasing PA levels in rural communities with limited access to PA resources. Study used school to implement a walking program. Choice of setting for walking: environmental benefits of program included safe, convenient, inexpensive, and weather independent. Indoor walking programs make it so that you can walk non-stop (don't have to stop for cars, curbs, holes in the ground) Being able to choose level or pace of walk (i.e., Schools with a variety of routes and corridors, or a mixture of stairs and hallways, were considered particularly attractive.) Existing school insurance policies were sufficient for the walking programs, and no additional staff time was reportedly required at any of the schools
Robinson, 2014 [138]	8 rural counties: 4 in Alabama (AL), 4 in Mississippi (MS). Population: Perry County, AL (*N *= 10,591), 71.7% high school graduate or higher, 68.0% Black; Bullock County, AL (*N *= 10,914), 72% high school graduate or higher, 69.5% Black; Sumter County, AL (*N *= 13,763), 75.4% high school graduate or higher, 73.6% Black; Wilcox County, AL (*N *= 11,670), 72.4% high school graduate or higher, 72.2% Black; Grenada County, MS (*N *= 21,906), 76.3% high school graduate or higher, 41.8% Black; Humphreys County, MS (*N *= 9,375), 64.7% high school graduate or higher, 74.4% Black; Panola County, MS (*N *= 34,707), 72.8% high school graduate or higher, 48.9% Black; Yazoo County, MS (*N *= 28,065), 74.4% high school graduate or higher, 57.1% Black	Rural Active Living Assessment (RALA)	Recreation facilities; schools sidewalks; streets			**X**	This study assessed neighborhood characteristics, town programs and policies, and town-wide facilities for PA in the eight counties with-in the AL Black Belt and the MS Delta. Only one of the eight communities had a town policy requiring bikeways or pedestrian walkways in new public infrastructure projects (town policies domain). School policy domain scores, indicating public access to school recreational facilities after school and/or a late transportation option for children who participated in after school activities, ranged from 15 to 30 (of 30) and mean scores of 19 in Alabama and 23 in Mississippi. Most communities had some type of school program, with domain scores from 0 to 30. The rural communities (*n *= 8) evaluated in this study lacked a strong base of policies and programs to support PA, particularly outside of policies and programs related to local schools
Sánchez, 2014 [87]	9 key informant interviews and 2 focus groups	Key informant interviews and focus groups on barriers, facilitators, and recommendations regarding school nutrition and PA policy implementations	Schools		**X**	**X**	Key informants reported school food policy compliance including soft drink and candy vending machines elimination and the existence of free and reduced cost meals. The success of policies was attributed to compliance from administration, principals, and parents. Financial resources to implement programs was noted as a major facilitator to PA. Lack of time for PA in the school day because of competing programs and insufficient understanding of written policies by staff and parents were noted as barriers to PA. Administrators noted off-campus vendors who regularly circumvented nutrition policy as a challenge. Both the key informants and the students shared challenges of adhering to the “50/50 rule” where 50% of school-sponsored fund-raisers follow healthy food guidelines. Even though both districts had adopted written wellness policies, key informant data showed a widespread lack of specific policy knowledge among superintendents, principals, wellness coordinators, and other representatives, with little shared understanding of who was responsible for carrying out and enforcing school health and wellness policies. A frequently cited PA barrier was the limited, formal PE requirement (one middle and one high school PE class)
Sanderson, 2002 [162]	61 Women; 67% some education past high school, 100% African American	Focus groups about PA personal, psychological, and biological barriers: social and physical environment support and barriers, policy, and cultural factors were also discussed	Childcare; sidewalks; streets; workplace			**X**	Policy facilitators to PA: speed breakers for traffic. Policy barriers to PA: work environments, not long enough lunch break to be active, day care centers closing early, safety concerns—lack of sidewalks, streetlights, and presence of unleashed or stray dogs, violence
Schetzina, 2009 [88]	23 teachers (96% female), 12 parents (92% female), and 19 fourth grade out of 97 students (58% female)	Open-ended questions about perceptions of school nutrition, PE, family and community involvement with children's eating and PA	Schools		**X**	**X**	Parents, teachers, and students identified barriers to PA in schools, including teachers withholding PA from students as punishment and providing insufficient time for PA due to focus on academia. Participants identified perceived barriers to healthy eating at school including a lack of rules and poor supervision over healthy choices within school nutrition services. According to school policy, students are not required to choose a fruit or vegetable as a part of their meal from the cafeteria
Shah, 2019 [132]	36 participated in interviews; 13 in panels (rural, urban, and midsize)	A semi-structured interview guide that explored program leaders’ experiences delivering Supplemental Nutrition Assistance Program-Education	Health department		**X**		Rural programs’ limited funding had an influence on programming. Those working with limited budgets reported constraints in conducting their work. Several leaders reported focusing heavily on financial feasibility when planning. Rural settings also challenged the implementation of certain interventions and evaluation tools. Rural leaders must carefully consider local politics, however there are sometimes limited opportunities to establish relationships with political leaders. In rural areas, politics had an important role in determining acceptable programs, framing interventions, and the number of opportunities a health department might have to engage partners
Sharkey, 2008 [111]	3.6% not reported educational background, 30.7% < 7th grade, 31.6% 7th-11th grade, 31.4% high school graduate	Colonia Household and Community Food Resource Assessment that included demographic questions, participation in food and nutrition assistance programs, access and mobility status, food stores, participants’ perception of community food environment, household food security, and alternative food sources	Food assistance programs		**X**		The women from the *Colonias* households mentioned that participation in federal food assistance programs decreased food insecurity. Participants who used supermarkets as main outlets for purchasing foods reported declined food insecurity. Increased distance to regularly visited supermarkets was associated with food insecurity
Sherry, 2008 [89]	8 food service managers; 4.1% had not completed high school, and 49.3% had no more than a high school diploma or GED	School Health Index (SHI) Module 4: Nutrition Services Score Card was given to the food service manager at each school	Schools		**X**		All schools provided breakfast and lunch, half the schools had a nutrition program to decrease sodium and fat content in school meals, the other half had programs to lowered sodium and fat that were partially in place
Smith, 2009 [112]	57 total rural residents; *n *= 36 in Iowa: 33% male, 67% female; 16.6% American Indian, 2.7% Asian, 64% Caucasian; Education: less than 8th grade:19%, 8th-12th grade: 22%, high school diploma/GED: 33%, associate degree: 8.3%, Master or PhD completed: 2.7%. Minnesota (*n *= 21): 38% male, 62% female; 4.25% American Indian, 81% Caucasian, 9.5% Hispanic; Education: less than 8th grade: 9.5%, 8th-12th grade: 57%, high school diploma/GED:19%, associate degree: 4.25%	Focus groups consisting of key informants and professionals working in food assistance programs	Food assistance programs		**X**		Rural residents perceived the benefits they received from food assistance programs as fewer than those received by urban residents. Food assistance programs were viewed as an important aspect of the food system, including Women, Infants and Children (WIC), Senior Meal Program, School Lunch Program, Food Stamp Program, and food shelves/pantries
Thomson, 2019 [144]	10 rural towns (excluded 2 towns because they exceeded recommended populations for rural area)	Rural Active Living Assessment (RALA) and the Community Park Audit Tool	Schools; streets; recreation facilities			**X**	Of the rural towns assessed (*n *= 10), the mean town program score was 3 (SD = 6) out of 30 possible points, town policy score was zero (0) for all towns, mean school program score was 3 (SD = 5) out of 30, mean school policy score was 14 (SD = 13) out of 30, and the total mean was 19 (SD = 18) out of 100 possible points. High scores indicate the town's-built environment was more conducive to PA with factors composing public/private recreation, bikeways/walkways required, public access to recreation facilities and late bus options, and walking and safe routes to schools
Thomson, 2019 [145]	12 towns	Rural Active Living Assessment (RALA) to assess PA features in communities; The Program and Policy Assessment tool assesses PA programs and policies in towns; The Town-Wide Assessment tool assesses towns’ physical characteristics	Schools; streets			**X**	In total, 615 street segments were measured in 12 rural towns. The median length of the street segments was 0.19 miles (mean = 0.22, SD = 0.14). Neighborhood walkability safety features (sidewalks, buffers, shoulders, signage, connectivity) may play a role in PA. All segments had flat terrain and the most prevalent land use purposes were residential (98%), followed by open spaces (74%), public/civic (34%), and commercial (27%). Almost three-fourths of the street segments did not have any sidewalks (69%), sidewalk buffers or defined shoulders (73%), signage (crosswalk, pedestrian, or children at play) (69%), or posted speed limits (74%). Only 13% of segments had sidewalks on both sides. However, 90% neighborhood street segments received high ratings for walkability and aesthetics. The state of MS has adopted a Complete Streets policy that can help create more walkable communities. However, Mississippi has less than 11 such policies at local or regional levels, no combined bicycle and pedestrian master plan (or two stand-alone plans), and less than 15% of its schools participate in Safe Routes to School programs
Turner, 2019 [90]	< 400 students: 32%, 400–699 students: 36.9%, 700–999 students: 16.8%, ≥ 1,000 students: 13.1%; ≤ 75% eligible for free or reduced-priced meals: 56.2%, > 75% eligible for free or reduced-priced meals: 41.3%; Urban/suburban: 79.2%, Rural/township: 19.8%; 58.4% elementary school, 14% middle school, 20.3% high school; < 75% Latino: 69.5%, ≥ 75% Latino: 28.9%,	Administrative data for School Breakfast Programs and National School Lunch Program (NSLP) participation claims obtained through public records requests to the California Department of Education	Schools		**X**		Participation in USDA’s School Breakfast Program (SBP) increased an average of 3.48% and 5.79% for National School Lunch Program (NSLP). For breakfast, rural areas were the least likely to use a provision compared to urban and suburban schools (44.4%; χ2 = 196.68, *p* < .001); the same pattern was evident for lunch (41.5%; χ2 = 191.05, *p* < .001). By 2016–2017, adoption rates had also become higher for eligible schools in rural/township areas versus urban/suburban areas. Schools with fewer than 400 students had a lower availability of Summer Nutrition Programs (SNP) than larger schools. When eligible schools adopted provisions, participation rates increased an average of 3.48 percentage points for breakfast and 5.79 points for lunch the following year. Provision adoption was more common at larger schools, had predominantly Latino students, and were rural
West, 2013 [98]	8 stakeholders including elected officials, Parks and Recreation representatives, chamber of commerce, and the school board	Interviews and Common Community Measures for Obesity Prevention (COCOMO) to evaluate recommendations regarding the most winnable implemented strategies and perceptions of barriers and facilitators	Food retail; schools; parks; recreational facilities		**X**	**X**	Stakeholders identified conflicts between policies that would interfere with the private sector (e.g., public funding to subsidize supermarkets) and noted a lack of funding opportunities to support healthy strategies. However, stakeholders did identify the need to increase PA through PE classes or extracurricular activities and the acceptability of school-based obesity prevention strategies
Wallace, 2019 [91]	1,844 adults, children, and adolescents	Physical Activity Resource Assessment (PARA) to conduct recreational site audits and identify communities’ needs and strengths. Intercept surveys to assess healthy eating behaviors	Churches; schools		**X**	**X**	There was a strong focus on promoting healthy food choices in all 4 counties. The availability of healthy food preparation equipment catalyzed the adoption of nutrition-related policy changes in 6 churches, where they replaced some foods with healthier options (e.g., fried chicken replaced by grilled chicken). Two school systems agreed to implement a policy that allowed students to bring water bottles into the academic setting after C3 provided water bottle refilling stations. In Lake County, the Coordinated School Health representative implemented a change in school policy that led to banning unhealthy food as rewards to students. 9 churches allowed non congregational use of their indoor and outdoor facilities and 11 schools allowed use of their playground equipment to community members
Yousefian, 2009 [155]	Three town case studies were assessed: townscape audits using a modified version of the Irvine-Minnesota audit tool.3 rural low-income communities; *n *= 6 focus groups with *n *= 84 total participants (school-aged children; 54.8% female, majority white); *n *= 15 key informant interviews with rural town planners, school personnel, directors of recreation, law-enforcement, local business owners, public health officials, and parents	Townscape audits, youth focus groups, and key informant interviews	Schools; streets; sidewalks			**X**	Participants identified safety concerns including stray animals, unknown adults, and traffic safety. Sidewalks, crosswalks, and signals were inconsistent and only in downtown areas, which are determined in part by Maine state policy. Policies related to zoning were seen as a barrier to PA, a need for mixed-use zoning near town centers would help rural communities to be more activity friendly. State policies that governed locations of schools were found to affect PA, since schools near downtown areas tend to have increased access to after school programming and activities. A lack of transportation to and from community and school-based PA opportunities and facilities was a primary barrier identified, including the lack of late-school buses. Schools were identified as the primary place for policy considerations that could impact PA (in school and afterschool)

*U.S. *United States, *BF* Breastfeeding, *HE *Healthy Eating, *PA* Physical Activity

**Table 3:** Grey literature about breastfeeding, healthy eating, and physical activity policies in rural U.S. settings (*n *= 46)

Source		Behavior			Results
Author Title	Setting(s)	BF	HE	PA	Policy
Alaska (AK) Department of Health and Social Services, Division of Public Health AK Breastfeeding Initiative. [50]	Hospitals			**X**	Hospitals that implemented CDC’s Ten Steps to Successful Breastfeeding included the Yukon Kuskokwim Health Corporation in Bethel, AK and PeaceHealth Ketchikan Medical Center in Ketchikan, AK
Bishop Tucumcari, New Mexico (NM) HEAL MAPPSTM Community Report. [75]	Playgrounds; schools		**X**	**X**	Support for healthy eating described as food assistance programs (not specified), improved salad bars at school, adhering to federal guidelines, and providing free breakfast, lunch, and snack to Tucumcari students. Supports for PA included community use of track and high school gyms during the summer (joint/shared use effort)
California (CA) Breastfeeding Coalition Baby Friendly Hospital Initiative. [66]	Hospitals	**X**			Designated Baby-Friendly Hospitals included: Barstow Community Hospital; George L. Mee Memorial Hospital in King City; Marshall Medical Center in Placerville; Northern Inyo Hospital in Bishop; Robert E. Bush Naval Hospital in Twentynine Palms; Sutter Amador Hospital in Jackson; Tahoe Forest Hospital in Truckee; Ventura County Medical Center Santa Paula Hospital; and Weed Army Community Hospital in Fort Irwin
Case Bonanza, Oregon (OR) HEAL MAPPSTM Community Report 2015. [172]	Churches			**X**	Shared use/joint use efforts supporting PA included access to gym and classes (Zumba) at community churches
Center for Health Equity, Education, and Research Mississippi (MS) Champs Hospital. [67]	Hospitals	**X**			Hospitals that implemented the Baby Friendly Hospital Initiative included: Baptist Memorial Hospital in Columbus; Anderson Regional Medical Center in Meridian; Baptist Memorial Hospital in Oxford; Baptist Memorial Hospital in New Albany; Highland Community Hospital in Picayune; King's Daughters Medical Center in Brookhaven; Merit Health Madison in Madison County; Merit Health River Oaks in Flowood; Merit Health Woman's Hospital in Flowood; North MS Medical Center in Tupelo; North MS Medical Center in Amory; South Sunflower County Hospital in Indianola; and the Southwest MS Regional Medical Center in McComb
Center for Health Equity, Education, and Research CHEER Champion of the Week: Blackfeet Nation Community Breastfeeding Team. [68]	Hospitals; workplace	**X**			The Poka-Friendly Community project assists the Blackfeet Community Hospital with maintaining Baby-Friendly status; the “Breastfeeding in the Workplace” policy for employees was successfully adopted by the Browning Public-School District, Montana (MT) on May 25, 2016
Center for Health Equity, Education, and Research Chippewa Cree Tribe Increases Support for Breastfeeding. [51]	Hospitals	**X**			A hospital in Havre, MT, described as the main delivering hospital for Rocky Boy’s Reservation, was noted as on the pathway to becoming Baby Friendly
Foundation for Healthy Communities Celebrating Our Collective Progress: 10 Years of Healthy Eating and Active Living in New Hampshire (NH). [125]	Schools; streets; childcare		**X**	**X**	New municipal complete streets policies, healthy food and PA standards in early care centers, and Safe Routes to School programs were among the many changes made
Fredericks Kalama, Washington (WA) HEAL MAPPSTM Community Report. [101]	Recreational facilities, schools, food assistance programs; food retail		**X**	**X**	Shared use/joint use efforts supporting PA included track and field and school policies to allow community use of school recreational facilities. Track field and school policies allow community use of school recreational facilities. Supports for healthy eating include ensuring fresh fruits and vegetables are available at grocery stores and facilitating access to food assistance programs
Halverson Molalla, OR HEAL MAPPSTM Community Report 2015. [102]	Food assistance program; community centers; food retail; parks; schools; streets		**X**	**X**	HEAL MAPPS™ (Healthy Eating Active Living: Mapping Attributes using Participatory Photographic Surveys) tool was used to assess barriers to HE that included issues of access and transportation
Harden Clatskanie, OR HEAL MAPPS™ Community Report 2015. [114]	Food assistance programs; parks; recreation facilities		**X**	**X**	HEAL MAPPS™ (Healthy Eating Active Living: Mapping Attributes using Participatory Photographic Surveys) is used to discuss maps and photographs that support HE in communities. Increasing access to number of food assistance programs was one of the ways to achieve HE in Clatskanie, OR
Harden Rainier, OR HEAL MAPPS™ Community Report 2015. [120]	Schools; food retail		**X**	**X**	Shared use/joint use efforts supporting PA included the school district in this offering community access to resources like the pool, track, and trails on school properties. Policies to increase participation in buying clubs from local farmers and farmer’s market, through a co-op service. Increasing knowledge of efforts and resources via advertisement on website or other media
High5 For Mom & Baby High 5 for Mom & Baby Facilities. [53]	Hospitals	**X**			High 5 For Mom & Baby provides resources and a framework to help birth centers improve health outcomes for breastfeeding women and their infants. The ten High 5 for Mom & Baby practices are based on the WHO/UNICEF Ten Steps to Successful Breastfeeding—evidence-based practices proven to increase breastfeeding success and reduce racial and ethnic disparities. Participating facilities complete a voluntary and self-reported evaluation of their adherence to the High 5 for Mom & Baby practices
Hill Building a Successful Community Coalition–University Partnership at the Arizona (AZ) – Sonora Border. [76]	Schools		**X**	**X**	Coalition members implemented the CDC School Health Index to identify the strengths and weaknesses of the school’s health promotion policies and programs. Health education was reintroduced into the schools’ curriculum and specific time was scheduled for PE in the elementary schools. In addition, the Food Services Director made changes to the cafeteria food that was served to Douglas Unified School District students and the nutrition committee drafted the school nutrition policy. The nutrition policy set standards for elementary through high school and included no deep-fried foods, no candy incentives or sales, and appropriate portion sizes. The policy was unanimously passed by administration and governing board and was adopted in March 2005 (a year in advance of the deadline)
Holston Implementing Policy, Systems, and Environmental Change Through Community Coalitions and Extension Partnerships to Address Obesity in Rural Louisiana (LA). [103]	Streets; food retail, parks, school		**X**	**X**	The implemented PSE strategies varied somewhat by community but included park revitalization, stenciling play spaces, healthy retail initiatives, Smarter Lunchroom initiatives, community and school gardens, initiating and supporting farmers markets, downtown beautification projects, Complete Streets demonstrations, and Complete Streets rural plan development
Indiana (IN) State Department of Health State Breastfeeding Plan & Baby-Friendly Hospital Initiative. [52]	Hospitals, Community	**X**			The following hospitals/medical centers achieved Baby Friendly status: Logansport Memorial Hospital (Logansport, IN); Henry Community Health (New Castle, IN); Pulaski Memorial Hospital (Winamac, IN); Parkview Whitley Hospital (Columbia City, IN); Reid Health Hospital (Richmond, IN); Saint Joseph Regional Medical Center – Plymouth (Plymouth, IN); Schneck Medical Center (Seymour, IN)
Ishdorj et al Are rural infants benefiting from WIC food package rule changes? Breastfeeding and infant feeding behaviors. [74]	Food assistance programs	**X**			Rural mothers had a significant, small increase in breastfeeding initiation post WIC changes (pre/post: 65.9%, 66.5%). Rural mothers increased selection of the fully breastfeeding package (pre/post: 11.6%, 16.4%—4.8% increase, *p* < 0.01. Partial breastfeeding and fully formula choices decreased slightly, although only the former was statistically significant (pre/post partial: 18.4%, 14.9%—3.5% decrease, *p *< 0.01)
Kansas (KS) Breastfeeding Coalition, Inc Communities Supporting Breastfeeding. [54]	Hospitals	**X**			The hospitals participating in the High 5 for Mom and Baby program include: Memorial Hospital in Abilene; Arkansas City Republic County Hospital in Belleville; Coffeyville Regional Medical Center in Coffeyville; Newman Regional Health in Emporia; St. Luke's Cushing Hospital in Leavenworth; Hospital District #1 of Rice County in Lyons; Community HealthCare System in Onaga aSource also lists those noted above
Kearny County Hospital (KS) Fast Facts: A community report on your investment in Kearny County Hospital. [121]	Food retail, local food producers, hospitals		**X**		Examples of healthy eating policy updates in Kearny County is Shorty’s Too & Club who pledged to offer healthy options
Louisiana (LA) Department of Health Gift Designated Facilities. [55]	Hospitals	**X**			The Gift Designated facilities included: North Caddo Medical Center in Vivian; Our Lady of the Angels Hospital in Bogalusa; Bayne-Jones Army Community Hospital in Fort Polk South; Beauregard Health System in De Ridder; Byrd Regional Hospital in Leesville; Minden Medical Center in Minden; Morehouse General Hospital in Bastrop; Lakeview Regional Medical Center in St. Tammy Parrish
Missouri (MO) Department of Health and Senior Services "Show-Me 5″ Initiative. [65]	Hospitals	**X**			The following hospitals adopted the MO “Show-Me 5” Hospital Initiative: Hannibal Regional Hospital in Hannibal; Fitzgibbon Hospital in Marshall; Lake Regional Health System in Osage Beach; Mercy Hospital Lebanon in Lebanon; Cox Medical Center Branson in Branson; Poplar Bluff Regional Medical Center in Poplar Bluff; Hedrick Medical Center in Chillicothe
National Institute for Children’s Health Quality Being Small Has Advantages for Hospitals Implementing the Ten Steps. [56]	Hospitals	**X**			United Memorial Medical Center in Batavia is one of 13 hospitals participating with the National Institute for Children’s Health Equity in the New York State BF Quality Improvement in Hospitals Learning Collaborative
National Physical Activity Society Stories from Small Towns: Hebron, Nebraska (NE). [154]	Schools, trails			**X**	On Walking Wednesdays, 180 elementary kids are dropped off at the courthouse to walk a mile up to the school. The focus has become eight-foot-wide trails, where it’s easy to travel peacefully by foot or pedals
National Physical Activity Society Stories from Small Towns: Soap Lake, WA. [165]	Streets			**X**	Soap Lake revitalization included a Complete Streets policy and bike lanes on the state highway running through the town
NM Breastfeeding Task Force Baby-Friendly Hospitals and Clinics. [57]	Hospitals	**X**			The following medical centers achieved Baby-Friendly Hospital status: Gallup Indian Medical Center in Gallup; Gila Regional Medical Center in Silver City; Northern Navajo Medical Center in Shiprock; Zuni Comprehensive Health Center in Zuni
Pollack Porter Promoting Physical Activity with Temporary Street Closures. [166]	Streets			**X**	Findings revealed that Play Streets are a good way to get kids active in rural communities. Each of the Play Streets included a variety of activities using temporary play spaces and equipment, with inflatables being the most popular activity and where children seemed to be most active. We also found that in rural communities, streets are not always the most accessible places for Play Streets to be implemented, considering that there are fewer streets and those streets often are major thoroughfares through town
Powers-Hammond Connell, WA HEAL MAPPS™ Community Report. [146]	Schools			**X**	Shared use/joint use efforts supporting PA included school policies that allow facilities to be used by community members including the new cross-country track and recreational programs offered through the Parks and Recreation department
Prevention Institute Northeast Iowa (IA) Food & Fitness Initiative. [156]	Schools; streets			**X**	The Northeast IA Food & Fitness Initiative developed a Safe Routes to School Program with a walking bus (group of students walking to/from school with responsible supervisors), remote drop-off areas, and bike rodeos (teaching how to ride bikes)
Reis-Reilly Breastfeeding in the community. Addressing disparities through policy, systems, and environmental change interventions. [72]	Workplace, libraries, local businesses	**X**			Center for Health Equity developed a workplace support policy and assisted libraries and local businesses in becoming breastfeeding friendly
Robert Wood Johnson Foundation Garrett County, Maryland (MD): A Community Holding Hands to Bridge its Divide. [163]	Sidewalks, trails, neighborhoods			**X**	Local governments are working on plans to build sidewalks and recreational trails, linking historic sites, low-income housing, and town centers
Robert Wood Johnson Foundation Hardworking Rural Community Taps a Deep Well of Hope. [160]	Trails			**X**	To encourage active transportation like walking and biking in the Klamath Falls area, OR, in 2016 the City Council approved an urban trail master plan created by the county and OR Department of Transportation
Robert Wood Johnson Foundation Hearing from Everyone on Health. [126]	Healthcare		**X**	**X**	The Governor of OR enacted a law that required a new system for managing federal dollars to include low-income residents’ medical costs, which allowed for a collaborative approach to addressing health in these areas
Robert Wood Johnson Foundation Manistique, MI: 2013 RWJF Culture of Health Prize. [100]	Streets; schools		**X**	**X**	Manistique has a non-motorized transportation plan to get everyone in the community out walking and biking safely and has implemented a Safe Routes to School project to encourage kids to walk to school regularly. It also developed a coordinated school health plan to provide students with healthy breakfasts, farm-fresh foods, and quality PE that features fun activities like archery, snowshoeing, and Zumba classes
Rural Health Information Hub Strengthening the Workforce to Improve Pregnancy Outcomes in Rural Areas. [58]	Hospitals	**X**			Mercy Hospital was one of two Critical Access Hospitals in the state to be designated as a Baby-Friendly facility
Rural Health Information Hub Albert Lea Blue Zones Project. [77]	Schools		**X**	**X**	Project schools adopted the following policy initiatives to teach healthy lifestyles to students: healthy snack carts, water bottle filling stations, smarter lunchroom program, grab-and-go breakfasts, indoor recess kits, standing desks. 7 schools participated with all students K-12
Rural Health Information Hub Coordinated Approach to Child Health in the Upper Peninsula (CATCH-UP). [136]	Schools; streets			**X**	The Western Upper Peninsula Department, MI implemented Safe Routes to School to create safe, convenient, and fun opportunities for children to bike and walk to and from schools. These safe route projects were incorporated into city and township master plans. Committees of administration, teachers, upper-grade students, parents, and community members furthered the Safe Routes to School model to build support for more policy, systems, and environmental changes within the community
Rural Health Information Hub Granville Greenways, North Carolina (NC) Walkable Community. [157]	Streets; trails			**X**	As a part of the Granville Greenways project to promote walking and biking trail implementation (greenways), the county has a greenway master plan, five county municipalities have created comprehensive pedestrian plans and two county municipalities have bicycle plans. The plans help guide community decision-making and provide necessary background for funding requests
Safe Routes Partnership Rural Communities: Making Safe Routes Work. [137]	Schools; streets			**X**	Schools that implement the Safe Routes to School program utilize remote drop offs. At the remote drop off location, students congregate as they are dropped off, and then walk the rest of the way to school in a walking school bus with an adult volunteer. The program is organized to allow students who take the school bus to participate too – if students bring in a permission slip, the bus driver will drop them off at the remote drop off location to walk with the others. To include students who aren’t able to participate in these options, the school also allows students who are driven to school to contribute to classroom participation goals by walking around the track
Smart Growth America Complete Streets Work in Rural Communities. [167]	Streets			**X**	There is a 2009 resolution for Complete Streets in Woodstock, NY. De Soto, MO’s 7,000 residents will benefit from a 2008 ordinance requiring a Complete Streets approach. And the city of Manistique in MI’s Upper Peninsula recognizes how Complete Streets support economic outcomes
Smart Growth America The Best Complete Streets Initiatives of 2017. [164]	Streets			**X**	Warsaw has implemented five of the seven key implementation steps that lead to lasting, successful Complete Streets initiatives: Adopt a policy, revise plans & processes, offer trainings, engage the community, and implement projects
Tah Four IHS Hospitals Complete Baby-Friendly Re-designation. [59]	Hospitals	**X**			The Indian Health Service launched the Baby-Friendly Hospital Initiative in 2011. Four of the 10 IHS hospitals designated as Baby-Friendly recently achieved re-designation. Baby-Friendly hospitals focus on increasing breastfeeding initiation and duration using quality improvement processes to improve breastfeeding rates through new maternity care and infant feeding practices
Tingey Wells, NV HEAL MAPPS™ Community Report. [107]	Food assistance program, food retail, recreation facilities, schools		**X**	**X**	HEAL MAPPS™ (Healthy Eating Active Living: Mapping Attributes using Participatory Photographic Surveys) was used to discuss supports and barriers for PA and HE. Supports for PA include youth recreation sports programs, a community center, an intertribal council gym, Chimney Rock golf course, outdoor trails and parks, playgrounds, and some sidewalks. School recreation programs and facilities include recess and after school sports programs. Barriers to PA included lack of sidewalks or lack of maintaining sidewalks, affordability of existing exercise facilities, safety concerns at public parks and trails. Supports for HE includes hot lunch served at school, vending machines that include healthy snacks, local produce available through farmers and co-op, healthy food retail options such as Subway. Barriers to HE includes no hot lunch at the middle and high school, and lack of local food production
United States Department of Agriculture The Pennsylvania SFSP Rural Area Eligibility Pilot Evaluation. [78]	Schools		**X**		Pre-existing Summer Food Service Program rural sponsors also administered sites meeting 40% threshold. There was a lot of fluctuation over year in and out of SFSP for rural sites. The number of urban sites increased, and number of rural sites decreased, but the number of new SFSP sites serving rural areas meeting the 40% threshold increased each year
United States Department of Agriculture Report to Congress: The NE Rural Area Eligibility Determination Pilot for the Child and Adult Care Food Program. [108]	Food assistance programs		**X**		The Child and Adult Care Food Program was modified by policymakers to increase rural children eligibility by applying the Public Law 108–265 (2004) to include 40% of school children below 185% poverty level instead of regular program eligibility that require 50% of school children to be below 185% poverty level. Little success in encouraging providers from remote areas to join CACFP but kept many providers in for longer periods
University of MO Extension MO Workplace Wellness Award. [73]	Workplace	**X**	**X**	**X**	The worksite wellness program provides awards to business based on the following criteria to designate bronze, silver, or gold regarding supportive environments for breastfeeding. Gold status extends beyond BF to include supports for healthy eating and PA (have an area to exercise onsite, policy in place for healthy food choices during meetings, celebrations, cafeteria/vending)
Voices for Healthy Kids, American Heart Association Strong Coalition Enabled City of Pryor Creek to Pass First Complete Streets Ordinance in Oklahoma (OK). [168]	Streets			**X**	The City of Pryor Creek, OK successfully implemented a Complete Streets ordinance No. 2016–01 by agreeing to approve the Ordinance unanimously

*U.S. *United States,* BF* Breastfeeding, *HE* Healthy Eating, *PA* Physical Activity

**Table 4:** Graduate Research about Breastfeeding, Healthy Eating, and Physical Activity Policies in Rural U.S. Settings (*n* = 3)

First Author, Year	Population	Measures	Setting(s)	BF	HE	PA	Policy
Glagola Dunn, 2018 [129]	26 church leaders. 38% male and 62% female. Primarily white (77%) or Black participants (19%), with 4% identified as Native American	Interviews questions focused on health concerns for the congregation and community; activities for youth and food and beverage availability; who was perceived to have decision making power for health-related activities for youth; and features of the church or mission that align with addressing childhood obesity	Churches		**X**	**X**	A HE policy included church leaders emphasizing stories about dietary choices of Biblical figures to youth. One PA policy required church social events to include active games or other PA opportunities for youth
Martin, 2018 [79]	46,356 students (no other information provided)	De-identified data from the Department of Health and 2013–2014 county free and reduced-price school lunch program participation from the Annie E Casey foundation	Schools		**X**		Free and reduced school lunch participation was a significant predictor of overweight/obesity (*p *< 0.001), even when controlling for rurality. Rural counties had significantly higher rates of participation in the free and reduced-price school lunch program (53%) compared to non-metro (36%) and metro (34%) neighborhoods
Menking-Hoggatt, 2017 [60]	55 mothers (34 in the intervention and 21 in the control groups). In both groups, mothers were predominantly white (94% or 95%)	Phone questionnaire to collect mothers’ demographics and other qualitative data about breastfeeding experiences, skin-to-skin contact, and birth memories	Hospitals	**X**			A skin-to-skin contact (SSC) intervention (i.e., maternal-infant contact postpartum) improved any BF at 6 weeks (82.4% vs. 61.9%), at 3 months (73.5% vs. 52.4%), and at 6 months (44.1% vs. 42.9%) compared to control (not statistically significant). The SSC intervention was also found to improve exclusive BF at 6 months (73.5% vs. 57.1%) and 3 months (64.7% vs. 33.3%), compared to control. At 6 months, exclusive BF was less than control group (20.6% vs. 23.8%; not statistically different) Noted barriers included lack of support for BF outside the hospital, low (50% of population) access to WIC, and no statewide BF goals in West Virginia

*U.S.* United States, *BF* Breastfeeding, *HE* Healthy Eating, *PA *Physical Activity

**Table 5:** Methods and Tools for Assessing Breastfeeding, Healthy Eating, and PA Policy Factors in Rural Communities

Breastfeeding		Healthy Eating		Physical Activity
**Method/Tool**	^**Sources**^	**Method/Tool**	^**Sources**^	**Method/Tool ** ^**Sources**^
Data from Baby-Friendly USA [61, 64] Maternity Practices in Infant Nutrition and Care (mPINC) survey [63] Employee Perceptions of Breastfeeding Support Questionnaire [70] Hospital Breastfeeding Policies: Self-Appraisal Pre-Assessment [62] Interviews [29, 71] Phone questionnaire [60]		Administrative data for School Breakfast Programs and National School Lunch Program (NSLP) [90] Bridging the Gap food code/policy coding forms to identify healthy food outlets [99] Common Community Measures for Obesity Prevention (COCOMO) Assessment [98, 131] Community-Based Policy and Environmental Supports for Healthy Eating and Active Living Survey [113, 134] Community Healthy Living Index’s Early Childhood Program Assessment Tool [124] Comprehensive Participatory Planning and Evaluation Model (CPPE) informed workshops [110] Demand-stimulating policies assessed as SNAP coverage (SnapCov), measured as the proportion of the population below poverty receiving SNAP benefits. Number of SNAP recipients and poverty rate (PovRate) used to construct SnapCov come from the U.S. Bureau of Census Small Area Income and Poverty Estimates (SAIPE) [116] Free and reduced-price school lunch program participation from 2013–2014 by county was obtained from the Annie. E Casey foundation [79] Household survey to assess access to food and the food system [109] Intercept surveys [91] Interviews and/or Focus Groups [80, 87, 88, 98, 104, 105, 109, 112, 119, 129, 130, 132, 135] Michigan Food Service Director Farm-to-School Survey [83] National Survey of Community-Based Policy and Environmental Supports for Healthy Eating and Active Living (CBS HEAL) [133] Nutrition and Physical Activity Environment Assessment Tool [82] Nutrition and Physical Activity Self-Assessment for Child Care (NAP SACC) [122, 123] Observational protocol to observe the National School Lunch Program provided in schools [92] Photovoice [94] School records of participation in School Breakfast Program [84] The Adams County Food Policy Council Questions [118] Process evaluation measure to assess School Breakfast Participation [86] School Environment and Policy Survey [104–106, 135] School Health Index (SHI) [89] Tool for Health and Resilience in Vulnerable Environments (THRIVE) [128] Web-based survey to locate Sodium Reduction Strategiesin hospitals [127] Wellness School Assessment Tool (WellSAT) [96]		Behavioral Risk Factor Surveillance (BRFSS) Survey [149, 153, 170] Community Healthy Living Index (CHLI) Early Childhood Program Assessment Tool [124] Community Audits and Surveys to assess farmers 'market shopping frequency, shopping at various markets, awareness of markets, access to markets, and barriers to and facilitators of use of farmers' markets [131] Community Health Assessment and Group Evaluation (CHANGE) Tool [161] Comprehensive Participatory Planning and Evaluation (CPPE) informed workshops [110] Irvine-Minnesota audit tool (modified for rural context) [155] Interviews and/or focus groups [80, 87, 88, 104, 105, 115, 129, 130, 135, 147, 150, 151, 155, 162, 171] National Health Interview Survey [149] National Survey of Community Based Policy and Environmental Supports for Healthy Eating and Active Living [113, 117, 152, 159, 169] Nutrition and Physical Activity Self-Assessment for Child Care (NAP SACC) [122, 123] Photovoice [94] Rural Active Living Assessment (RALA) [138, 139, 142, 144, 145, 210] School Environment and Policy Survey [104–106, 135] Policies collected from all Utah school district [93] Survey that asked about plans municipalities implemented and projects, program policies to improve walking and bicycling [158] Collected updated pedestrian plans through conducted web searches, the North Carolina Department of Transportation Division of Bicycle and Pedestrian Transportation plan library, jurisdictions when necessary for follow-up, and through a listserv of North Carolina planners [141]

*PA* Physical activity

## Discussion

### Summary of findings

This scoping review identified policy supports for breastfeeding, healthy eating, and/or PA in rural areas of the U.S., as part of a larger project to compile information on existing PSE change approaches encompassing these behaviors. Results show that policy initiatives for breastfeeding included changes to implement certain standards or practices, increase access to resources, and improve culture or norms mainly in hospitals and workplaces. Policy initiatives for healthy eating included increasing access to healthy foods, reducing the financial burden of purchasing healthier foods, requiring programs or initiatives to promote healthy eating, and improving food assistance programs. Policy initiatives for PA focused on joint or shared use agreements, Safe Routes to Schools efforts, coordinated plans or master plans for the community or county to implement or improve greenways, trails, and transportation options, coordinated school health plans, and childcare or school PA standards. Methods and tools to assess policy changes related to breastfeeding, healthy eating, and PA mostly included interviews or focus groups, surveys, assessment tools, or administrative data. Findings from this scoping review can be used to develop policy and surveillance recommendations to promote breastfeeding, healthy eating, and PA in rural communities that have been historically under-resourced.

### Contributions to current literature

This scoping review adds to existing research which compiles and reviews policy approaches to improve rates of breastfeeding [34, 173–177], healthy eating [24, 178–185], and PA [21, 186–194]. For breastfeeding promotion policies, our findings align with existing reviews that demonstrate the need for tracking policies to protect, promote, and support breastfeeding in hospital, workplace, and community settings [34, 173–176]. For healthy eating promotion policies, our results confirmed findings from past reviews showing that policies addressing transportation and access barriers and bridging partnerships between retail outlets or schools and local food producers may be effective for improving healthy eating among rural residents [24, 178–185]. Schools were also identified as important settings for healthy eating promotion, and many sources cited policies to increase access to federal or state level child nutrition programs [78, 79, 83, 90, 93], or to include nutrition standards on the school level that increase availability of healthy foods [75, 76, 85, 92, 93]. Despite these findings, existing literature points to a lack of policy examples and related literature in small or isolated rural areas experiencing the greatest healthy eating disparities [178, 183]. For PA promotion policies, our results aligned with findings from past reviews showing that existing policy approaches to improve PA are being implemented in rural areas but additional research is needed to demonstrate their effectiveness, especially when considering the lag that often exists between implementation and research and the impracticality of randomized control trials in these settings [21, 186–194]. Nonetheless, existing research supports the implementation of PA policy in school/childcare, workplace, and community (e.g., parks, recreational facilities, streets) settings to address barriers related to PA resource access and safety within PA environments [21, 186–194]. This often included policies to ensure pedestrian and bike infrastructure plans were developed and implemented [113, 136, 138, 139, 141, 144, 145, 156, 159, 163–165, 167–169], and that policies existed to ensure active transportation to school was possible [100, 103, 136, 137, 143, 144, 154]. Despite alignment with past reviews focused explicitly on policy approaches for breastfeeding, healthy eating, or PA promotion, existing reviews do not specifically focus on rural environments, explore multiple behavioral settings, or include grey literature sources. Therefore, our results fill an important gap in the literature by compiling policy-focused health promotion strategies in under-resourced, rural areas [195].

### Implications for policy, practice, and research

Results from this scoping review have several implications for policy, practice, and research. To start, healthy eating [75–98, 104–106, 122–124], and PA [75, 82, 87, 88, 91, 93, 98, 100, 101, 104, 106, 113, 120, 122–124, 135–153, 155, 162] policies were often implemented in schools and childcare settings. Given schools and childcare centers are critical resources for rural communities and the number of healthy eating and PA policies in these settings, researchers and practitioners should explore potential policy surveillance mechanisms on the district/administrative level and track their effectiveness [196]. Rural schools and childcare settings may also benefit from partnerships with local health departments, Extension offices, and research institutions due to reduced capacity for dissemination, implementation, and evaluation of policy approaches.

For breastfeeding, results show that policies in hospitals and workplace settings are most common to encourage rural breastfeeding rates [50–65, 68–72]. Hospital adoption and implementation of breastfeeding-supportive policies was the most common factor related to breastfeeding [51–55, 57, 59, 61, 62, 64–68], although declines in the number of rural hospitals and healthcare workers may pose a unique challenge for rural hospital maintenance of these policy strategies. [50–65] Annual assessments in rural areas may be beneficial, and it is recommended that terminology captures the variety in strategy names identified in this review in addition to “Baby-Friendly” (e.g., “The Gift”,“High 5 for Mom & Baby”) and the level of progress in adopting full policy strategies (e.g., 8 out of 10 steps) [53, 55, 59, 61, 62, 64]. Policies to support breastfeeding in workplace settings could be a focus of surveillance efforts, since barriers exist for breastfeeding among lower income and impoverished workers [197, 198]. Moreover, existing information in support of this recommendation is primarily qualitative so additional quantitative assessments are needed [69–72].

For healthy eating, food assistance programs and food retail settings were identified as important for policy implementation [97–99, 102, 107–113, 115–119]. In response to these findings, we recommend that rural policy data collected by food assistance programs (e.g., SNAP-Ed, WIC) be leveraged for surveillance purposes. One potential solution is to use existing evaluation and data systems within state and local-level SNAP-Ed and Extension offices to monitor healthy eating promotion policy efforts that are not consistently published in peer-reviewed sources [199, 200]. Given the presence of both SNAP-Ed and Extension across rural America, tracking combined efforts is important and should be explored as a surveillance data source.

For PA, public settings like trails/paths [130, 149, 157–160], streets [100, 103, 113, 136–145, 157–159, 162, 164–167, 169], recreational facilities [140, 141, 149, 170], neighborhoods [131], and parks [113, 141] were found to be particularly important for policy efforts in rural areas. We recommend increased monitoring of policy implementation efforts and related adaptations in rural towns, including Safe Routes Partnerships, Complete Streets, shared or joint use, and town-level or school plans (e.g., trails, bike, pedestrian, transportation, master, coordinated school health plans) [137, 164, 167–169]. Additional school-based policy recommendations include tailoring policy work to each school, gaining support from policy makers and school administrators, and recognizing that schools located closer to the downtown areas have increased access to after school programming and activities [75–106, 135, 156]. In order to gain support of school administrators, it is evident that we need to do a better job sharing evidence with them on the academic benefits of PA, as is demonstrated through one quote by a superintendent, “What we continue to hear is ‘No Child Left Behind.’ I haven’t heard ‘Don’t leave fat kids behind.’ It’s about keeping kids academically fit.” (pg. S155) [135].

Overall, for policy initiatives across all three targeted behaviors of this review, there is a need to create infrastructure for data sharing across rural communities that includes open-source access, easy-to-use visualizations, and raw data. Rural organizations and stakeholders often lack capacity to access and analyze existing data to inform their work regarding breastfeeding, healthy eating, and PA. Providing easily accessible, publicly available data can enable local rural organizations to access relevant and actionable information to support and implement policy efforts [201–204]. Next, an open-source platform would allow stakeholders to easily share data and results given the difficulty in capturing this data independent of a system [205, 206]. This would in turn allow for improved surveillance of policy efforts in settings that are often overlooked in existing publications and reports, such as libraries, community gardens, health departments, local government, and faith-based organizations. Finally, many classification systems and definitions of “rural” are used in the sources identified for this scoping review. This ranged from standardized rural classification systems (e.g., RUCA, RUCC) to somewhat arbitrary descriptions (e.g., smaller population sizes, authors describing the study site as “rural”). Moreover, some sources met criteria for “rural” using the “Am I Rural?” tool, and did not meet criteria for rural a using a rural–urban typology (e.g., big cities; college towns; exurbs; middle suburbs; military posts; urban suburbs) [48]. Future research should employ more standardized rural definitions, and report the rural definition used.

### Strengths and limitations

This scoping review presents rural policy strategies used to encourage breastfeeding, healthy eating, and PA over a twenty-year period to inform public health approaches and has several strengths. First, this scoping review employed a scientific librarian to ensure our search terms were comprehensive, included training for those carrying out our screening efforts using strict protocols, and engaged multiple reviewers to identify review articles. Adding to this, we made sure to conduct our review using a pre-existing theoretical framework that allowed the research team to accurately review the body of literature and compile information on each article that related to PSE change approaches [207, 208]. The PSE change framework is increasingly used to address health behavior and outcome disparities and this review synthesizes the growing body of literature on this topic [207, 208]. Next, this scoping review provides a holistic understanding of rural-specific policy change approaches for improving breastfeeding, healthy eating, and PA from academic and non-academic sources. While some past research has conducted systematic reviews of peer-reviewed original research on rural policy regarding breastfeeding [34, 173–176], healthy eating [24, 178–185], and PA [21, 186–194], promotion, it is important to incorporate distinct findings from grey literature and dissertation research about rural health promotion policies.

This study also has limitations. First, the scoping review search is a bit dated, being carried out in 2020. However, as the data for this review were pulled from a comprehensive review (resulting from a comprehensive search strategy) that encompassed PSE approaches (rather than only policy), carrying out an updated search requires substantial resources for which the study team no longer has funding. Additionally, there were likely changes to the nature of the literature published post-2020, due to the COVID-19 pandemic and numerous federal approaches to improve food and nutrition outcomes, in particular. Updated reviews can be carried out for strategic purposes (e.g., to compare pre- and post-pandemic policy for breastfeeding, healthy eating, and PA promotion in rural areas); however, authors do not consider this limitation to threaten the value of the 122 studies synthesized here. Second, it should be acknowledged that PSE approaches were often overlapping and not necessarily distinct. As an example, many policy changes are related to the environment (e.g., establishing pedestrian master plans), required the implementation of the policy on a systems or organizational level (e.g., tracking of Complete Streets implementation), and then resulted in increased environmental supports (e.g., number of greenways/trails in the community) [209]. Despite this limitation, publishing separate scoping review papers that describe PSEs independently across the target behaviors (in preparation) is important for compiling PSE change approaches to promote health in rural America without overwhelming readers with the breadth of existing knowledge. Third, we did not integrate formal reliability checking during article selection or results synthesis phases or provide ratings of bias for selected articles. The scope and size of the project, combined with an expedited timeline during the early months of the COVID-19 pandemic, limited our ability to report these types of metrics; however, we adhered to a rigorous protocol for scoping reviews and held regular search and extraction meetings to ensure consistent adherence to the inclusion/exclusion criteria and results synthesis process across all team members [39]. Fourth, many articles did not list a clear rural definition or failed to designate study settings as rural, which may have limited our ability to include all existing research relevant to this scoping review. Despite this, we employed methods (e.g., using the “Am I Rural” tool for grey literature sources) to ensure as much existing knowledge on policy approaches relevant in rural areas were captured.

## Conclusions

This scoping review identified policy supports that encourage breastfeeding, healthy eating, and/or PA practices in rural American communities. Results from this comprehensive review of effective and empirically supported policy strategies can be used to inform future efforts to address low rates of breastfeeding, healthy eating, and PA in rural areas to address chronic disease disparities [39]. Given the identified policy strategies are already occurring in under-resourced rural settings, we recommend opportunities for novel surveillance of these indicators that move beyond individual behavior statistics to identify structural changes to make healthier choices the easier choices in the rural U.S.

## Data Availability

The complete search strategy and all data generated or analyzed from articles meeting inclusion criteria for this study as it pertains to this manuscript are included in this published article.

## Abbreviations

BF: Breastfeeding
CDC: Centers for Disease Control and Prevention
FAR: Frontier and Remote Area
HE: Healthy Eating
PA: Physical Activity
PRISMA-ScR: Preferred Reporting Items for Systematic Reviews and Meta-Analyses extension for Scoping Reviews
RUCA: Rural Urban Commuting Area
RUCC: Rural-Urban Continuum Codes
SNAP: Supplemental Nutrition Assistance Program
SNAP-Ed: Supplemental Nutrition Assistance Program - Education
U.S.: United States
UICs: Urban Influence Codes
WIC: Women, Infants, and Children
